# Pure Organic AIE Nanoscintillator for X‐ray Mediated Type I and Type II Photodynamic Therapy

**DOI:** 10.1002/advs.202302395

**Published:** 2023-07-09

**Authors:** Yuewen Yu, Lisha Xiang, Xuanwei Zhang, Le Zhang, Zhiqiang Ni, Zhong‐Hong Zhu, Yubo Liu, Jie Lan, Wei Liu, Ganfeng Xie, Guangxue Feng, Ben Zhong Tang

**Affiliations:** ^1^ State Key Laboratory of Luminescent Materials and Devices Guangdong Provincial Key Laboratory of Luminescence from Molecular Aggregates School of Materials Science and Engineering AIE Institute South China University of Technology Guangzhou 510640 China; ^2^ Division of Thoracic Tumor Multimodality Treatment and Department of Medical Oncology Department of Radiation Oncology Cancer Center State Key Laboratory of Biotherapy West China Hospital Sichuan University Chengdu Sichuan 610041 China; ^3^ Analysis and Testing Research Center East China University of Technology Nanchang 330013 China; ^4^ State Key Laboratory of Chemo/Biosensing and Chemometrics Hunan University Changsha 410082 China; ^5^ Department of Oncology and Southwest Cancer Centre Radiation Treatment Centre Southwest Hospital Third Military Medical University (Army Medical University) Chongqing 400038 China; ^6^ School of Science and Engineering Shenzhen Institute of Aggregate Science and Technology The Chinese University of Hong Kong Shenzhen Guangdong 518172 China

**Keywords:** aggregation‐induced emission, organic scintillators, photodynamic therapy, radiotherapy, X‐ray induced photodynamic therapy (X‐PDT)

## Abstract

X‐ray induced photodynamic therapy (X‐PDT) circumvents the poor penetration depth of conventional PDT with minimal radio‐resistance generation. However, conventional X‐PDT typically requires inorganic scintillators as energy transducers to excite neighboring photosensitizers (PSs) to generate reactive oxygen species (ROS). Herein, a pure organic aggregation‐induced emission (AIE) nanoscintillator (**TBDCR NPs**) that can massively generate both type I and type II ROS under direct X‐ray irradiation is reported for hypoxia‐tolerant X‐PDT. Heteroatoms are introduced to enhance X‐ray harvesting and ROS generation ability, and AIE‐active **TBDCR** exhibits aggregation‐enhanced ROS especially less oxygen‐dependent hydroxyl radical (HO^•−^, type I) generation ability. **TBDCR NPs** with a distinctive PEG crystalline shell to provide a rigid intraparticle microenvironment show further enhanced ROS generation. Intriguingly, **TBDCR NPs** show bright near‐infrared fluorescence and massive singlet oxygen and HO^•−^ generation under direct X‐ray irradiation, which demonstrate excellent antitumor X‐PDT performance both in vitro and in vivo. To the best of knowledge, this is the first pure organic PS capable of generating both ^1^O_2_ and radicals (HO^•−^) in response to direct X‐ray irradiation, which shall provide new insights for designing organic scintillators with excellent X‐ray harvesting and predominant free radical generation for efficient X‐PDT.

## Graphical abstract

A pure organic aggregation‐induced emission (AIE) nanoscintillator (**TBDCR NPs**) is developed for X‐PDT. **TBDCR NPs** could be directly excited by X‐ray to emit bright fluorescence and massively generate singlet oxygen and less oxygen‐dependent hydroxyl radicals in aggregate for type I and type II X‐PDT, which demonstrate excellent tumor eradication performance to radio‐resistant tumors both in vitro and in vivo.

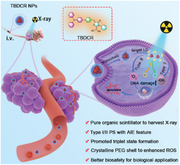

## 1 Introduction

Since Roentgen's discovery of X‐ray in 1895 for the first time, X‐ray have received widespread research interests in biomedical imaging and therapeutics.^[^ ^1^ ^]^ After more than 100 years of unremitting efforts by scientists, X‐ray‐mediated radiation therapy (RT) has been developed as one of the mainstream methods for clinical cancer treatments, which has been commonly employed to treat more than 50% of cancer patients.^[^ ^2^, ^3^, ^4^ ^]^ RT capitalizes on high‐energy ionizing radiation to damage cellular deoxyribonucleic acid (DNA) or radiolysis water to generate toxic reactive oxygen species (ROS). However, the high X‐ray dosage, unavoidable ionizing irradiation damage to healthy tissues and the tumor hypoxia‐related RT resistance have been major obstacles in RT‐based cancer therapy.^[^ ^5^, ^6^ ^]^ To date, numerous approaches have been employed to increase the therapeutic efficacy of RT, including oxygen delivery, increasing radiosensitivity, modulating tumor microenvironment (TME), and so forth.^[^ ^7^ ^]^ Nonetheless, the state‐of‐art strategy to increase RT performance is still using high‐Z metal‐based radiosensitizers such as Pt, Au or tantalum oxide nanoparticles with high X‐ray attenuation coefficients to increase the intratumoral ionizing irradiation.^[^ ^8^ ^]^ Recently, several radiosensitizers such as hafnium dioxide (HfO_2_) nanoparticles (NBTXR3) have been tested in clinical trials in Europe and US for treating hepatocarcinoma and other cancers.^[^ ^9^, ^10^ ^]^ Despite the potency of these inorganic radiosensitizers to increase the RT efficacy, a very high X‐ray dosages (50–70 Gy) is still required and the generation of RT‐resistance cannot be circumvented. Hence, more efforts are urgently needed to increase effectiveness of RT without RT‐resistance generation for tumor eradication.

Photodynamic therapy (PDT) is a minimally invasive method for clinical cancer treatment,^[^ ^11^ ^]^ with advantages of precise controllability, noninvasiveness, high spatiotemporal accuracy as well as negligible drug resistance, which shows great potential in oncotherpy.^[^ ^12^, ^13^, ^14^, ^15^, ^16^ ^]^ Generally, PDT generates cytotoxic ROS utilizing light, oxygen, and photosensitizers (PSs) to deliver direct therapeutic effect, which could also elicit immunogenic cell death to activate the antitumor immunotherapeutic response.^[^ ^12^ ^]^ PSs can be categorized into two classes, type I PSs produce free radicals like hydroxyl radicals (HO^•−^), superoxide radicals (O_2_^•−^), by electron transfer mechanism from triplet excited PSs, while type II PSs generate singlet oxygen (^1^O_2_) through the energy exchange process with surrounding oxygens.^[^ ^17^, ^18^ ^]^ The vast majority of developed PSs belong to type II PSs, while very recent advance suggests that type I PSs are less oxygen‐dependent and more promising for hypoxia tumor treatment, benefited from intracellular superoxide dismutase (SOD)‐mediated disproportionation.^[^ ^19^ ^]^ In this regard, designing effective type I PSs for PDT has attracted great research interests recently.^[^ ^11^, ^12^, ^13^ ^]^ However, most of them (also including type II PSs) are excited by visible or near‐infrared (NIR) light where the limited light penetration depth due to light scattering and tissue absorption has greatly hampered their applications.^[^ ^20^, ^21^ ^]^ Although, utilizing multiphoton‐excitation or upconversion technique could also increase the penetration depth, the light penetration is still very superficial (<1 cm),^[^ ^22^, ^23^ ^]^ and the curative impact of PDT on deep tumors is far from satisfactory.

In recent years, X‐ray mediated PDT (X‐PDT) that combines the merits of deep tissue penetration and minimal drug resistance has attracted great research interests.^[^ ^7^, ^15^, ^24^, ^25^ ^]^ X‐PDT usually requires a inorganic scintillator as the energy transducer to convert X‐ray to visible light and subsequently excite neighboring PSs via a two‐step energy transfer (EnT) process.^[^ ^26^, ^27^, ^28^, ^29^ ^]^ Generally, current scintillation systems commonly used for X‐PDT include anchoring organic PSs at scintillator surface or constructing MOFs with heavy metal units and photosensitizing ligands.^[^ ^31^, ^32^, ^33^, ^34^ ^]^ However, combining these inorganic scintillators and organic PSs not only complicates material synthesis, but also undoubtedly results in tremendous energy loss from the inefficient two‐step EnT processes.^[^ ^30^ ^]^ On the other hand, organic scintillators which could be directly excited by X‐ray to emit photons have recently been reported by Huang, Liu and others.^[^ ^35^ ^]^ Organic scintillators could generate 25% singlet excitons and 75% triplet excitons under direct X‐ray irradiation,^[^ ^36^ ^]^ which is very promising for X‐PDT as ROS is generated from triplet excitons, but such possibility has been less explored. To the best of our knowledge, there is only one report of pure organic scintillator for X‐PDT, which is unfortunately a planar type II PS whose antitumor performance will be hampered by the hypoxia tumor microenvironment.^[^ ^36^ ^]^ Moreover, the vast majority of those reported PSs (for both type I and type II PSs) for PDT and X‐PDT have planar molecular structures, and they tend to form aggregates with strong π‐π stacking‐related aggregation‐caused quenching (ACQ) effect.^[^ ^37^, ^38^, ^39^, ^40^, ^41^, ^42^ ^]^ Such notorious ACQ effect results in unavoidable fluorescence quenching and low ROS efficiency for these PSs at aggregated states or at high concentrations in anaerobic tumor microenvironments, seriously affecting therapeutic efficacy of X‐PDT and PDT.^[^ ^11^ ^]^ As opposed to the ACQ effect, fluorogens with aggregation‐induced emission characteristic (AIEgens) display negligible fluorescence in the molecular state but show magnified fluorescence in aggregate as a result of the restriction of intramolecular motion (RIM) that blocks the nonradiative pathway.^[^ ^43^, ^44^ ^]^ Additionally, the inhibited molecular motions of the AIEgens in the aggregate could promote the radiative decay and intersystem crossing (ISC) process, offering the possibility of increased fluorescence and ROS generation.^[^ ^45^ ^]^ Therefore, it is crucial for creating pure organic AIE scintillators that can generate type I ROS under direct X‐ray irradiation for effective X‐PDT to solve all the above serious problems, as they could effectively generate triplet excitons, produce less‐oxygen dependent free radicals, and avoid ACQ compromised ROS generation in aggregate for ultimate tumor treatment.

**Scheme 1:**
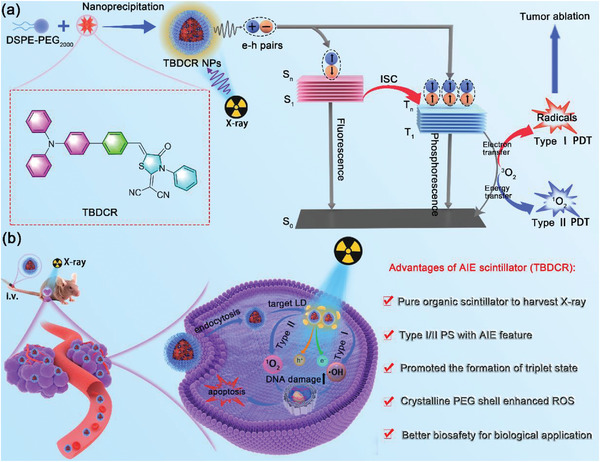
Schematic illustration of the structure and preparation of TBDCR NPs and its application in X‐PDT. a) Preparation of organic TBDCR NPs followed by ^1^O_2_ and radical ROS generation under direct X‐ray irradiation. b) Application of TBDCR NPs on fluorescence imaging‐guided X‐PDT cancer therapy.

In this work, we report a pure organic AIE nanoscintillator (**TBDCR NPs**) that profoundly generates ^1^O_2_ and HO^•−^ under direct X‐ray irradiation to realize effective X‐PDT (**Scheme** **1**). The introduced heteroatoms (oxygen, nitrogen and sulfur atoms) in **TBDCR** not only enhances the n‐π* transitions and accelerates ISC process to generate more long‐lived triplet state excitons, but also effectively increases X‐ray energy absorption and harvesting capability. Moreover, **TBDCR** shows efficient energy transfer and electron transfer processes after excitation that are beneficial for ^1^O_2_ and HO^•−^ generation. With the iconic AIE moiety triphenylamine (TPA) as the electron donor, **TBDCR** showed aggregation enhanced fluorescence, triplet exciton formation and ROS generation. **TBDCR** was further encapsulated into lipid‐polyethylene glycol (PEG) nanoparticles with sizes of ≈3.8 nm that is beneficial for tumor accumulation, deep tumor penetration and body clearance. Most importantly, **TBDCR NPs** exhibited a unique PEG crystalline shell, which leads to a rigid microenvironment and a much more compact packing for **TBDCR**, resulting in further improved ROS generation. Intriguingly, **TBDCR NPs** showed massive generation of ^1^O_2_ and higher toxic HO^•−^ under both light excitation and direct X‐ray irradiation, without the aid of inorganic energy transducers. Thanks to the ^1^O_2_ and HO^•−^ induced PDT and deeper tissue penetration ability of X‐ray, **TBDCR NPs** demonstrated excellent tumor eradication against HeLa cancer cells (Hela) as well as radio‐resistant HelaR tumors both in vitro and in vivo. This work of designing X‐ray activatable AIE nanoscintillator circumvents the limitations of poor penetration depth, low hypoxia tolerance and compromised ROS generation in aggregates for conventional PDT. Our design evidences the enormous potentials of type I AIE organic nanoscintillators in X‐PDT and shall pave new insights for further development of novel PSs as well as nanoscintillators for cancer theranostics.

## 2 Results and Discussion

### 2.1 Synthesis and Characterization of TBDCR

**Figure 1:**
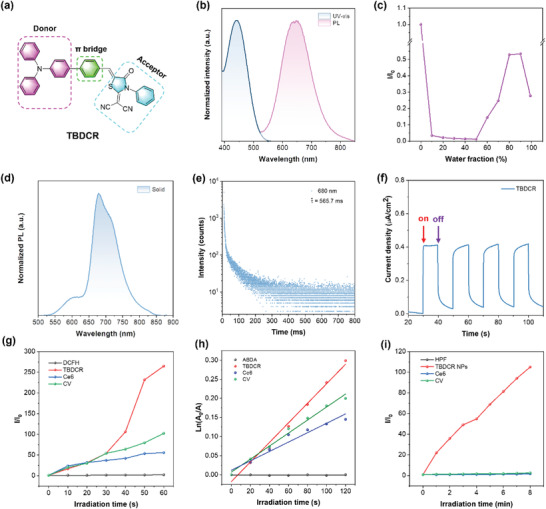
a) Chemical structure of TBDCR. b) Normalized absorption and PL spectra of TBDCR in THF. c) Plot of PL intensity versus water fraction of TBDCR in the THF/H_2_O mixtures. d). PL spectra of TBDCR in solid state. e) Time‐resolved decay profile of TBDCR (in solid state) at emission wavelength at 680 nm. f) Photocurrent responses of the TBDCR. g) Access of total ROS production with the PL enhancement of DCFH (50 µM) for different PSs (10 µM) upon the irradiation of white light (50 mW cm^−2^). h) Access of ^1^O_2_ generation with the decomposition of ABDA (50 µM) for different PSs (10 µM) upon the irradiation of white light (50 mW cm^−2^). i) Access of HO^•‐^ generation with PL enhancement of HPF (10 µM) for different PSs (10 µM) upon the irradiation of white light (50 mW cm^−2^).

It is particularly important in molecular design to improve the ability of materials to absorb X‐ray and to promote ISC process, thereby improving the generation of ROS under direct X‐ray irradiation. It has been reported high‐atomic number elements such as bromine atoms are beneficial for X‐ray absorption and transition.^[^ ^35^, ^36^ ^]^ In this work, oxygen, nitrogen, sulfur atoms were introduced to **TBDCR** to increase X‐ray energy harvesting capability, which also contribute to enhance the n‐π* transitions and promote ISC process to boost triplet state excitons.^[^ ^46^ ^]^ A famed AIE active group, TPA was employed as the electron donor because it possesses both non‐planar structure and powerful electron donating capability. 2‐(4‐Oxo‐3‐phenylthiazolidin‐2‐ylidene)malononitrile (DCR) moiety which contains heteroatoms (O, N, and S) was employed as an electron‐withdrawing group, and a benzene ring was selected as a π bridge to increase the π conjugation length and to promote highest occupied molecular orbital (HOMO)‐lowest unoccupied molecular orbital (LUMO) separation to reduce the valance electron repulsion for a better ISC process.^[^ ^15^ ^]^ Finally, as shown in **Figure** **1**a, the donor‐π‐acceptor (D‐π‐A) structured AIEgens (**TBDCR**) was obtained from the Suzuki coupling and Knoevenagel reaction with a high yield of 85%. The detailed synthetic routes of **TBDCR** were displayed in Scheme S1 (Supporting Information), and the chemical structures of all synthesized compounds were confirmed by nuclear magnetic resonance (^1^H NMR, ^13^C NMR), and high‐resolution mass spectrometry (HRMS) (Figure S1–S7, Supporting Information).

The optical characteristics of **TBDCR** were initially investigated in tetrahydrofuran (THF) by ultraviolet‐visible spectroscopy and photoluminescence (PL) spectroscopy. As displayed in Figure 1b, **TBDCR** has an absorption maximum at ≈443 nm and an emission peak at 653 nm, respectively, with a large Stokes shift of 210 nm. **TBDCR** possesses a very high molar absorption coefficient of 2.67 × 10^4^ M^−1^ cm^−1^ (at 443 nm) (Figure S8, Supporting Information), suggesting its excellent light harvesting capability that is advantageous for ROS generation. The AIE properties of **TBDCR** was then studied by measuring PL spectra in THF/water mixtures with increased water fractions (*f*_w_) to induce aggregate formation. As indicated in Figure 1c; Figure S9 (Supporting Information), the fluorescence intensity gradually reduced when increasing *f*_w_, reaching a minimum fluorescence intensity at *f*_w_ = 50%, which should be caused by the strong intramolecular charge transfer (ICT) feature of **TBDCR** as water possesses a higher polarity. However, further increasing *f*_w_ results in amplified emission intensities along with aggregate formation, which should be attributed to that the AIE effect competes over ICT effect. In addition, **TBDCR** powder showed distinct bright‐orange red fluorescence under a 365 nm UV lamp, further indicating that **TBDCR** possess excellent fluorescence in solid state and its AIE tendency (Figure S10, Supporting Information). Instead, the absolute fluorescence quantum yields (η) of **TBDCR** in solid state and THF solution were determined to be 38.1% and 17.6%, respectively, with the AIE effect (*α*_AIE_) value of ≈2.2. The absorption as well as emission spectra in solvents with various polarity were performed to study its ICT property. As shown in Figure S11 (Supporting Information), the absorption maxima of **TBDCR** in these different polar solvents are in the range of 440 to 470 nm, and it showed a very weak fluorescence in the more polar solvents like methanol and dimethyl sulfoxide, in sharp contrast to those in less polar solvents. Intriguingly, **TBDCR** showed an approximately 160 nm red‐shift in emission peak from hexane (low‐polar solvent) to dichloromethane (high‐polar solvent), which further suggests the strong ICT characteristics of **TBDCR**.

As introducing heteroatoms could boost triplet exciton formation, the phosphorescence‐related optical properties of **TBDCR** was further evaluated. Initially, the solid‐state fluorescence spectrum of **TBDCR** was measured. Interestingly, as displayed in Figure 1d, two emission peaks are presented at 608 and 680 nm, respectively. The emission peak at 608 nm should be the solid‐state fluorescence emission, which matches the solid‐state fluorescence (orange red) of **TBDCR** in Figure S10b (Supporting Information). The emission peak at 680 nm was attributed to the solid‐state phosphorescence of **TBDCR**, which was delightfully confirmed by time‐gated photoluminescence spectrum where a sharp PL peak at 680 nm was observed at a delay time of 0.1 ms (Figure S12, Supporting Information). In addition, the time‐resolved phosphorescence decay curve of the corresponding emission peak (680 nm) revealed an ultralong lifetime of 565.7 ms (Figure 1e), further confirming the phosphorescence nature and promoted triplet excitons for **TBDCR** powder, facilitating a better ROS production ability. It is also worth noting that **TBDCR** could also generate a high photocurrent of 0.4 µA cm^−2^ under light irradiation (Figure 1f), suggesting its electrons and holes generation and separation feature that is beneficial for free radical production through electron separation and transition processes. In addition, time‐dependent density functional theory (TD‐DFT) calculations (B3LYP/6‐31G (d, p)) were conducted for a deeper understanding of excellent ROS generation capability of **TBDCR**. As displayed in Figure S13a (Supporting Information), HOMO of **TBDCR** mainly concentrated at TPA moiety, while the LUMO are mainly located on the DCR moiety and the π bridge with an extensive HOMO‐LUMO separation. Such a large HOMO‐LUMO separation helps to reduce the repulsion of valance electrons and result in an extremely small S_1_ and T_1_ bandgap (ΔE_ST_) of 0.116 eV for **TBDCR** (Figure S13b, Supporting Information), which greatly promotes the ISC process and hence ROS generation. Collectively, these results reveal the excellent triplet exciton generation and efficient charge separation capability and suggest the promise of **TBDCR** to be an effective especially type I PSs.

Encouraged by the aforementioned excellent results, the ROS generation ability of **TBDCR** upon white light irradiation was then accessed. 2′,7′‐Dichlorodihydrofluorescein (DCFH), 9,10‐anthracenediyl‐bis(methylene) dimalonic acid (ABDA), dihydrorhodamine 123 (DHR 123), and hydroxyphenyl fluorescein (HPF) were selected as total ROS, ^1^O_2_, O_2_^•−^ and HO^•−^ indicators, respectively.^[^ ^47^ ^]^ As displayed in Figure 1g; Figure S14 (Supporting Information), the green fluorescence of dichlorodihydrofluorescein (DCF) was significantly enhanced in the presence of **TBDCR** within an ultra‐short exposure time (50 mW cm^−2^, 60 s). The fluorescence enhancement factor of DCFH was ≈265.2, suggesting the superior ROS generation capability of **TBDCR**. To study the AIE effect on ROS generation, the ROS of **TBDCR** at different *f*_w_ was evaluated. **TBDCR** showed negligible ROS generation at *f*_w_ of 0 and 40% (Figure S15, Supporting Information), which shall be due to that **TBDCR** is at its molecular state at relative low *f*_w_ and the excited energy was mainly non‐radiatively dissipated.^[^ ^48^ ^]^ In sharp contrast, the ROS generation was extremely significant when *f*_w_ rises to 99%, due to the aggregation‐enhanced ROS generation for AIE PSs.^[^ ^49^ ^]^ In addition, **TBDCR** showed a much better ROS generation than commercially available PSs, which is nearly 4.8‐ and 2.6‐fold higher than chlorin E6 (Ce6)^[^ ^50^ ^]^ as well as crystal violet (CV),^[^ ^51^ ^]^ respectively, as demonstrated by the DCF fluorescence enhancement factors.

Subsequently, ^1^O_2_, O_2_^•−^ and OH^•−^ generation of **TBDCR** exposure to white light was evaluated. ^1^O_2_ generation was assessed by decomposing ABDA upon light irradiation. As shown in Figure 1h; Figure S16 (Supporting Information), the absorbance of ABDA at 378 nm was significantly reduced in the presence of **TBDCR** upon white light illumination (50 mW cm^−2^, 120 s), which was reduced by ≈51.7%, while ABDA alone had almost no significant change. Ce6 and CV showed a minor effect on ABDA absorbance under the same conditions, with the ABDA absorbance reduced by only ≈12.5% and ≈14.7%, respectively. Moreover, ABDA decomposition rates as reflected by the slopes of ln(A_0_/A) versus irradiation time indicated that ^1^O_2_ generation efficiency of **TBDCR** was 2.1‐ and 15.1‐fold higher than Ce6 and CV (Figure 1h). Subsequently, the O_2_^•−^ generation capability of **TBDCR** under white light was evaluated by employing DHR 123 as the indicator. As illustrated in Figure S17 (Supporting Information), the fluorescence of DHR 123 was remarkably enhanced in the presence of **TBDCR** and white light irradiation (50 mW cm^−2^, 30 s). In contrast, the fluorescence of DHR 123 showed nonsignificant changes in the blank, Ce6 and CV groups receiving same light exposure. In addition, the DHR 123 fluorescence enhancement factor for **TBDCR** was determined to be ≈164.01, much higher than Ce6 (∼6.2) and CV (∼50.5), respectively, suggesting the excellent O_2_^•−^ generation capacity of **TBDCR** under white light irradiation. The production of OH^•−^ was evaluated with HPF. As shown in Figure 1i; Figure S18 (Supporting Information), under light irradiation, HPF itself showed hardly changed fluorescence signal, while the presence of **TBDCR** caused largely magnified HPF fluorescence intensity, and the HPF fluorescence enhancement factor for **TBDCR** with white light irradiation was determined to be ≈105.2. At the tested condition, Ce6 and CV only lead to a minimal amplification of HPF fluorescence signature, with the enhancement factors of ≈1.7 and ≈2.6, respectively. Such results clearly demonstrated that **TBDCR** has a proudly better OH^•−^ generating capacity than Ce6 and CV, which is almost 61.8‐ and 40.4‐fold higher than Ce6 and CV, respectively. The capability of **TBDCR** to generate ^1^O_2_ and OH^•−^ under white light irradiation was further conducted via electron paramagnetic resonance (EPR) with 2,2,6,6‐tetramethyl‐4‐piperidinol (TEMP) as well as 5‐dimethyl‐pyrroline n‐oxide (DMPO) as trapping agents for ^1^O_2_ and OH^•−^, respectively.^[^ ^52^ ^]^ The characteristic signals of ^1^O_2_/TEMP and OH^•−^/DMPO dramatically increased in the presence of **TBDCR** after light irradiation (Figure S19 and S20, Supporting Information), proving that **TBDCR** could efficiently manufacture both ^1^O_2_ and OH^•−^ when exposed to white light. In a word, **TBDCR** is an all‐in‐one PS that can efficiently generate type I and type II ROS especially in aggregate format, outperforming over Ce6 and CV, more promising for PDT.

### 2.2 Photophysical Properties and X‐Ray Mediated ROS Generation of TBDCR NPs

**Figure 2:**
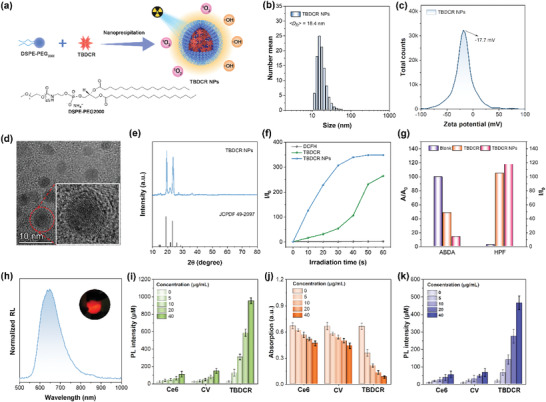
a) Schematic of the preparation of TBDCR NPs. b) Size distribution of TBDCR NPs. c) The ζ potential of the TBDCR NPs dispersed in deionized water. d) HR‐TEM image of TBDCR NPs (inset: enlarged HR‐TEM image). e) PXRD spectra of TBDCR NPs and PEG crystalline. f) Access of total ROS generation with DCF fluorescence enhancement for TBDCR aggregate (*f_w_* = 99% in THF/water mixture, 10 µM) and TBDCR NPs (10 µM) upon white light irradiation (50 mW cm^−2^). g) Access of ^1^O_2_ and HO^•‐^ generation with ABDA absorbance decomposition as well as HPF fluorescence enhancement for TBDCR aggregate (*f_w_* = 99% in THF/water mixture, 10 µM) and TBDCR NPs (10 µM) upon white light irradiation (50 mW cm^−2^), respectively. h) Normalized radioluminescence spectra of TBDCR aggregate (inset: photograph of TBDCR aggregate under direct X‐ray irradiation). i) Total ROS generation presented with DCF fluorescence enhancement for different PSs upon X‐ray irradiation (4 Gy) at different concentrations. j) ^1^O_2_ generation presented with ABDA (50 µM) decomposition for different concentrations of various PSs upon X‐ray irradiation (4 Gy). k) HO^•−^ generation presented with HPF (10 µM) fluorescence enhancement for different PSs upon X‐ray irradiation (4 Gy) at different concentrations. Data presented means ± SD, n = 6.

To endow **TBDCR** molecules with better water dispersion and biocompatibility, as demonstrated in **Figure** **2**a, the hydrophobic molecules were subsequently prepared into nanoparticles (NPs) named **TBDCR NPs** via an easily accessible nanoprecipitation approach with a biocompatible and amphiphilic copolymer 2‐distearoyl‐sn‐glycero‐3‐phosphoethanolamine‐*N*‐[methoxy(polyethylene glycol)−2000 (DSPE‐PEG_2000_) as the encapsulation matrix.^[^ ^53^, ^54^, ^55^, ^56^ ^]^ Dynamic light scattering (DLS) measurements indicated that the hydrodynamic diameter of **TBDCR NPs** was ≈18.4 nm (Figure 2b), which was preferable in tumor accumulation through enhanced permeability and retention effects.^[^ ^57^, ^58^ ^]^ The zeta potential of the **TBDCR NPs** was measured to be ≈−17.7 mV, affording them the excellent colloidal stability (Figure 2c). High resolution transmission electron microscopy (HR‐TEM) image indicated that the **TBDCR NPs** were distributed in a well‐defined and uniformly spherical morphology with monodisperse sizes of ≈3.8 nm (Figure 2d). Enlarged HR‐TEM images further revealed the mesh lattice strips from these **TBDCR NPs** (Figure 2d), which was also confirmed via the electron diffraction patterns from select area electron diffraction (SAED) result (Figure S21, Supporting Information), suggesting that **TBDCR NPs** readily formed nanocrystalline structures. The polycrystalline powder X‐ray diffraction (PXRD) spectrum of **TBDCR NPs** also indicated high crystalline phase of **TBDCR NPs**, and the crystalline was attributed be PEG (JCPDF 49–2097 from MDI Jade crystal database) shells (Figure 2e). Such a crystalline PEG shell provided a more rigid and dense intraparticle microenvironment for a more compact molecular packing, which theoretically further promotes ROS generation. To test our hypothesis, the photophysical properties of **TBDCR NPs** dispersed in water were subsequently studied. The absorption peak of **TBDCR NPs** was located at 458 nm, and the emission peak showed a slight blue shift to 620 nm as compared to those molecules in THF or aggregates in water (Figure S22, Supporting Information), which suggests a less polar and more compact intraparticle microenvironment. Moreover, **TBDCR NPs** exhibited a more profoundly enhanced ROS generation as compared to that of **TBDCR** aggregates (at *f*_w_ of 99% in THF/water mixture) by employing DCFH as the total ROS indicator under white light irradiation (Figure 2f; Figure S23, Supporting Information), and similar results were also observed in the terms of specific ^1^O_2_ and OH^•−^ generation performance (Figure 2g; Figure S24, Supporting Information), hinting that the formation of crystalline PEG shell is advantageous for enhancing ROS production capacity. In addition, crystalline PEG shells should also help improve these nanoparticles’ colloidal stability. Notably, the hydrodynamic diameter of **TBDCR NPs** barely changed in both water and 1×PBS buffer during 20 days storage at 4 °C (Figure S25, Supporting Information), and no noticeable aggregation was observed in the aqueous solution over 30 days storage, demonstrating excellent colloidal stability (Figure S26, Supporting Information).

The combination of **TBDCR NPs** and X‐ray will be more advantageous to increase X‐PDT effect as they possess amplified ROS generation and could be directed excited by X‐ray without intermediate inorganic scintillator and energy loss during a two‐step EnT. The radioluminescence (RL) of **TBDCR** was firstly studied, bright red fluorescence from **TBDCR** was clearly observed under X‐ray irradiation (Figure 2h), and it showed a RL spectrum ranging from 550 to 900 nm, confirming its organic scintillator nature. Subsequently, X‐ray mediated ROS generation was further studied with DCFH, ABDA and HPF as different ROS indicators. As shown in Figure S27 (Supporting Information), the fluorescence of both DCFH and HPF in the **TBDCR NPs** group increased dramatically upon X‐ray irradiation (4 Gy), and the absorbance of ABDA in the presence of **TNDCR NPs** group reduced significantly under X‐ray irradiation, suggesting that **TBDCR NPs** under X‐ray could effectively generate both ^1^O_2_ and OH^•−^. In addition, as manifested by DCF fluorescence enhancement factor in Figure 2i, the X‐ray (4 Gy) mediated ROS generation for **TBDCR NPs** (40 µg mL^−1^) was almost 9.1‐fold and 6.6‐fold higher than Ce6 and CV, respectively. With ABDA as ^1^O_2_ indicator, **TBDCR NPs** (40 µg mL^−1^ based on **TBDCR** concentration) leads to a ≈87% ABDA absorbance reduction with X‐ray dosage of 4 Gy, much better over Ce6 (≈30%) and CV (≈33%) (Figure 2j). As aforementioned discussion, type I PDT with the generation of HO^•−^ is less oxygen‐dependent and more favorable for hypoxia‐tolerant tumor treatment. Therefore, HO^•−^ generation under X‐ray irradiation was accessed with HPF as the indicator (Figure 2k). The fluorescence signal of HPF was significantly intensified in the presence of **TBDCR NPs** (40 µg mL^−1^) and X‐ray irradiation, with a fluorescence enhancement factor of ≈27.4. While, Ce6 and CV only led to much lower HPF fluorescence enhancement factors of ≈7.8 and ≈9.9, respectively. Moreover, the stability of **TBDCR NPs** under X‐ray irradiation was also evaluated, where the absorbance still maintained above 90% of original ones after exposure to X‐ray (8 Gy) (Figure S28, Supporting Information), suggesting the high stability of **TBDCR NPs** under X‐ray irradiation. In brief, our design of **TBDCR NPs** demonstrated excellent ROS generation and stability under direct X‐ray irradiation, which shall represent the first example of pure organic scintillator with type I ROS generation and hold great potentials in X‐PDT.

### 2.3 X‐PDT in Vitro

**Figure 3:**
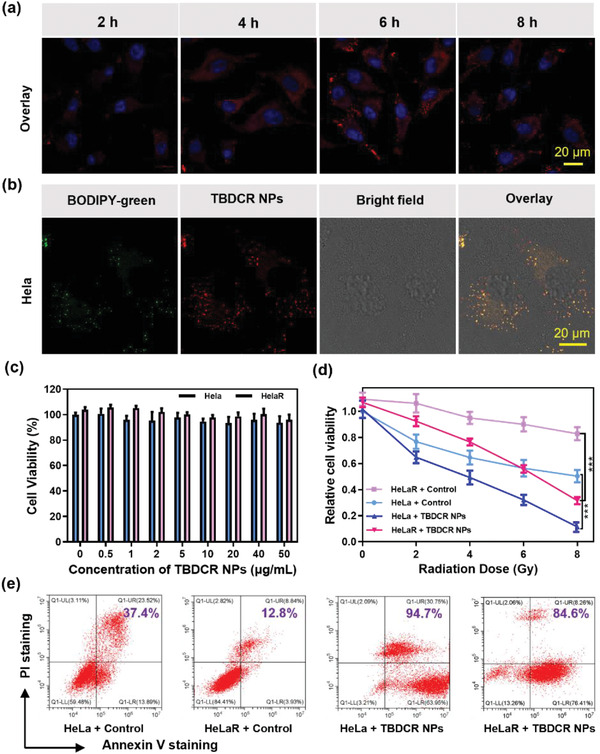
a) Confocal fluorescence images of cellular uptake of TBDCR NPs in Hela cells. TBDCR NPs: λ_ex_ = 405 nm, λ_em_ = 550–700 nm; Hoechest 33 342: λ_ex_ = 405 nm, λ_em_ = 430–470 nm, scale bars: 20 µm. b) Subcellular colocalization images of TBDCR NPs and BODIPY 493/503 in Hela cells. TBDCR NPs: λ_ex_ = 405 nm, λ_em_ = 550–700 nm; BODIPY 493/503: λ_ex_ = 488 nm, λ_em_: 500–515 nm. Scale bars: 20 µm. c) Cell viabilities of Hela/HelaR cells treated with TBDCR NPs at various concentrations under dark. d) Cell viabilities of TBDCR NPs (50 µg mL^−1^) treated Hela/HelaR cells after irradiating with X‐ray under various dosages. e) Cytotoxicity of TBDCR NPs in an Annexin V‐FITC/ propidium iodide (PI) apoptosis assay. Data presented means ± SD. n = 6, ***p < 0.001.

Given the strong ^1^O_2_ and HO^•−^ generation ability and NIR emission feature of **TBDCR NPs**, the X‐PDT related photoactivity experiments were conducted in vitro. Initially, the cellular uptake experiment of **TBDCR NPs** was performed with human cervical Hela cancer cells. As indicated in **Figure** **3**a; Figure S29 (Supporting Information), **TBDCR NPs** quickly accumulated inside HeLa cells after 2 h incubation with an intense and well‐defined red fluorescence signal in the cytoplasm, and the optimal cellular uptake was achieved at 6 h incubation. In addition, the fluorescence signal patten hints a possible lipid droplet (LD) staining possibility.^[^ ^59^ ^]^ The intracellular distribution of **TBDCR NPs** was evaluated using commercial organelle‐selective tracker BODIPY 493/503 to confirm the proposed conjecture. The observed red fluorescence of **TBDCR NPs** overlapped well with the green fluorescence of the lipid droplet tracker BODIPY 493/503 with a high Pearson's correlation coefficient of 0.87 (Figure 3b; Figure S30, Supporting Information), demonstrating excellent localization of **TBDCR NPs** at lipid droplet. LDs are newly recognized subcellular organelles that play important roles in lipid storage and metabolism, protein storage, membrane synthesis as well as molecular signaling.^[^ ^60^ ^]^ The direct ROS generation at LD may lead to a fast and effective LD oxidization and result in lipid peroxidation as well as ferroptosis to further promote therapy efficacy.^[^ ^60^, ^61^ ^]^ Therefore, we assume **TBDCR NPs** will be very effective in X‐PDT.

Furthermore, in vitro X‐PDT anticancer potency of **TBDCR NPs** was conducted using methyl thiazolyltetrazolium (MTT) assay by using HeLa cells and radiation‐resistant Hela cells (HelaR).^[^ ^6^ ^]^ As shown in Figure 3c, **TBDCR NPs** demonstrated negligible toxicity toward both Hela and HelaR cancer cells without light or X‐ray irradiation, at all tested concentrations, indicating their excellent biocompatibility. X‐PDT induced cell death was then evaluated at different X‐ray dosages. **TBDCR NPs** (50 µg mL^−1^ based on **TBDCR** concentration) effectively inhibited cancer cell proliferation in a radiation dosage‐dependent manner in both Hela and HelaR cancer cells, with the cell viabilities of ≈11.5% and ≈31.6% for Hela and HelaR, respectively (Figure 3d). However, the Hela and HelaR cells still maintain a high survival rate of ∼50.3% and ∼82.9% in the absence of **TBDCR NPs** even under a strong radiation dosage (8 Gy), which demonstrates the initial success of **TBDCR NPs** in X‐PDT. Notably, as shown in Figure S31 (Supporting Information), the excellent type I ROS generation capability of **TBDCR NPs** (50 µg mL^−1^) upon X‐ray irradiation (8 Gy) also granted its excellent X‐PDT performance under hypoxia condition (1% O_2_) that is comparable to those under normoxia conditions. Additionally, the more insights were provided on the X‐ray radiation‐caused cell death brought by **TBDCR NPs**. As shown in Figure 3e; Figure S32 (Supporting Information), the percentage of apoptotic cells in Hela and HelaR cells increased from 5.0% and 1.1% (blank control) to 94.7% and 84.6% after exposure to X‐ray (8 Gy), suggesting **TBDCR NPs** mediated X‐PDT could significantly increase cell apoptosis, even for radiation‐resistant HeLaR cancer cells. These findings showed a strong concordance with the MTT phototoxicity findings, indicating that the X‐PDT of **TBDCR NPs** can effectively induce tumor cell apoptosis and death promising for eradication tumor in vivo.

### 2.4 Mechanistic Study of X‐PDT

**Figure 4:**
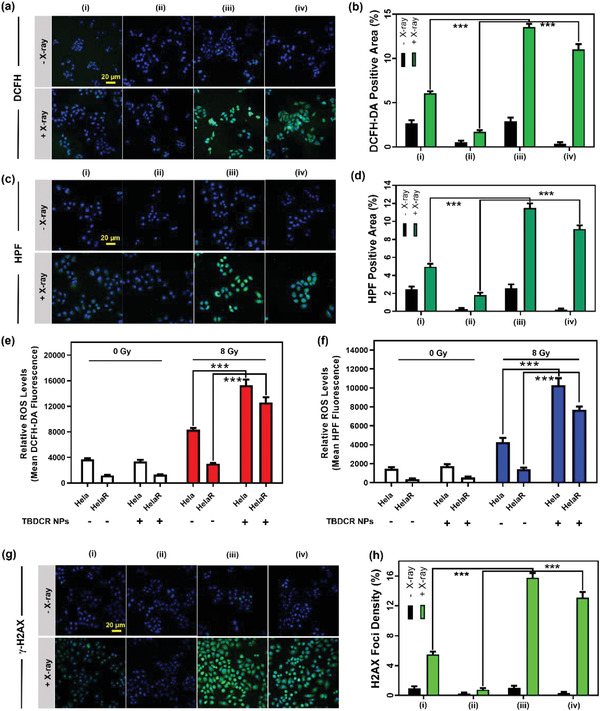
a) Confocal fluorescence images of DCFH‐DA stained Heal/HelaR cells treated with PBS or TBDCR NPs with and without X‐ray irradiation (8 Gy). Blue: DAPI; Green: DCFH‐DA. b) The relative DCFH‐DA positive area of each group in a). c) Confocal fluorescence images of HPF stained Heal/HelaR cells treated with PBS or TBDCR NPs with and without X‐ray irradiation (8 Gy). Blue: DAPI; Green: HPF. d) The relative DCFH‐DA positive area of each group in c). e,f) Respective flow cytometry measurement of intracellular ROS and HPF from different groups with and without X‐ray irradiation (8 Gy). g) Confocal fluorescence images of γ‐H2AX stained Heal/HelaR cells treated with PBS or TBDCR NPs with and without X‐ray irradiation (8 Gy). Blue: DAPI; Green: γ‐H2AX. h) The relative γ‐H2AX positive area of each group in g). Groups: i) Hela + control, ii) HelaR + control, iii) Hela + TBDCR NPs, iv) HelaR + TBDCR NPs. Scale bar: 20 µm. Data presented means ± SD. n = 6, ***p < 0.001.

The therapeutic mechanism of **TBDCR NPs** for X‐PDT was further explored. Initially, the intracellular ROS generation upon treatment with **TBDCR NPs** was investigated by employing 2′,7′‐dichlorodihydrofluorescein diacetate (DCFH‐DA), a reputable total ROS hypersensitive fluorescent probe.^[^ ^62^ ^]^ As displayed in **Figure** **4**a; Figure S33 (Supporting Information), the intracellular fluorescence intensities of DCF in both Hela and HelaR cells treated with **TBDCR NPs** after X‐ray irradiation (8 Gy) were significantly higher than their corresponding control groups, and DCF positive area was enhanced by ≈2.1 and ≈5.6 fold when compared to Hela and HelaR cells in X‐ray irradiation without **TBDCR NPs** incubation (Figure 4b), indicating that **TBDCR NPs** exhibited efficiently increased intracellular ROS levels through X‐ray irradiation. This establishes a solid basis for the subsequent apoptosis of tumor cells. In addition, the intracellular type I ROS production of **TBDCR NPs** was also studied by employing HPF as the HO^•−^ indicator. As illustrated in Figure 4c; Figure S34 (Supporting Information), the emergence of HPF fluorescence inside both **TBDCR NPs** treated HeLa and HelaR cancer cells upon X‐ray irradiation was clearly observed, with a ≈2.3 and ≈4.7 fold higher in HPF positive area that those only with X‐ray irradiation treatment, respectively, clearly demonstrating the production of highly toxic HO^•−^ inside cells (Figure 4d). Moreover, the flow cytometry was also employed to further analyze the intracellular ROS production capacity. The DCF positive cells for Hela and HelaR risen from 27.4% and 3.6% (blank control with only X‐ray irradiation) to 99.8% and 85.2%, respectively, after exposure to X‐ray (8 Gy) (Figure S35, Supporting Information). Notably, after **TBDCR NPs** and X‐ray radiation (8 Gy) treatment, the relative ROS levels in Hela and HelaR were ≈1.9 and 5.0‐fold higher than these only radiation groups (Figure 4e). In addition, after X‐ray (8 Gy) exposure, the HPF fluorescence positive percentages for Hela and HelaR respective increased from 28.2% and 1.9% (blank control with only X‐ray irradiation) to 98.3% and 71.4% (Figure S36, Supporting Information). Meanwhile, the Hela and HelaR treated with **TBDCR NPs** showed relative higher HO^•−^ levels that were ≈2.5 and 5.6‐fold stronger than these merely radiation groups (Figure 4f). All of these results agreed with the in vitro comparison of different groups both for the detection of total ROS and HO^•−^.

Generally, DNA damage is the most lethal damage caused by X‐ray.^[^ ^63^ ^]^ Moreover, highly reactive HO^•−^ can swiftly damage DNA in the proximity, which has a better effect on DNA damage than those with less reactive ROS.^[^ ^64^ ^]^ Thus, the DNA damage of cancer cells caused by **TBDCR NPs** mediated X‐PDT was investigated by immunofluorescence staining using γ‐H2AX, a famed double‐stranded DNA breaks marker.^[^ ^65^ ^]^ As displayed in Figure 4g; Figure S37 (Supporting Information), negligible DNA damage was observed in all the groups without X‐ray irradiation. However, the experimental groups incubated with **TBDCR NPs** under X‐ray irradiation showed stronger green fluorescence of γ‐H2AX than the control groups both in Hela and HelaR cells, with an enhanced γ‐H2AX positive area of ≈3.0 and ≈17.3 fold in Hela and HelaR cells as comparted to these groups with X‐ray irradiation only (Figure 4h), suggesting excellent X‐PDT effect of **TBDCR NPs** to cause increased DNA damage to both HeLa cells as well radiation‐resistant HeLaR cells. The much larger γ‐H2AX positive area difference between HelaR and HelaR + **TBDCR NPs** with X‐ray irradiation also manifests that X‐PDT could circumvent the RT‐resistance for a better RT outcome. All of these findings suggest that our AIE nanoparticles can cause significant enhanced intracellular ROS levels and DNA damage under X‐ray for improved tumor treatment performance.

### 2.5 In vivo Tumor Fluorescence Imaging and X‐PDT

**Figure 5:**
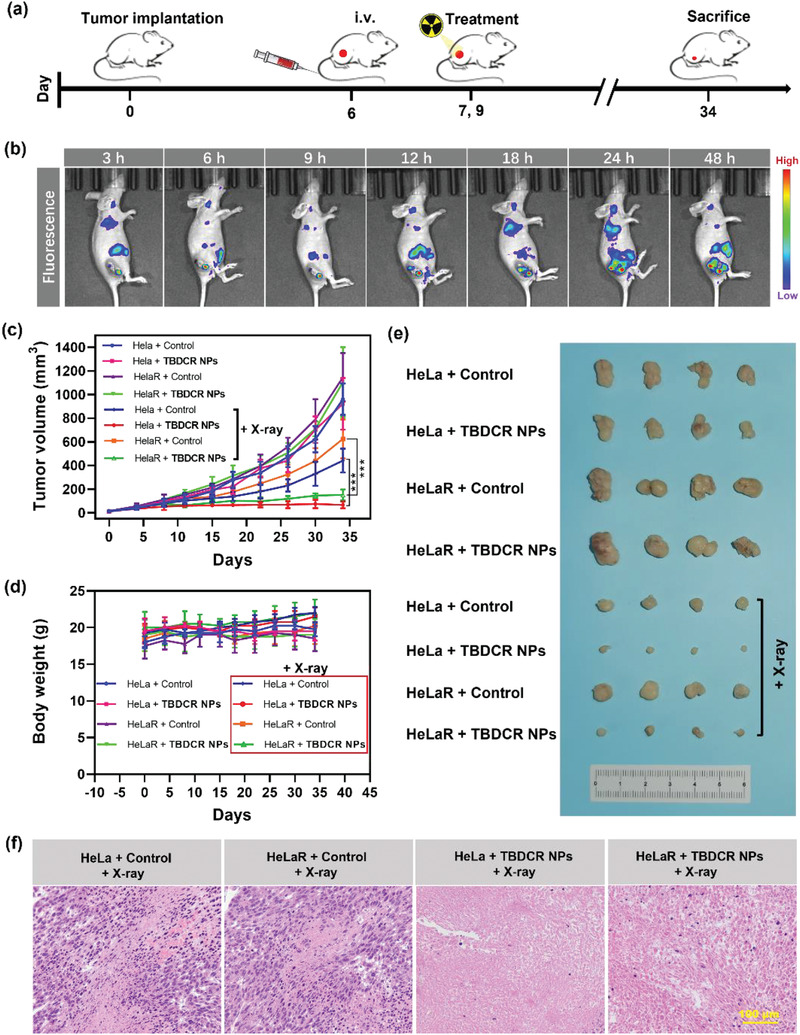
In vivo therapeutic efficacy of TBDCR NPs‐mediated X‐PDT. a) Schematic illustration of tumor implantation and TBDCR NPs‐mediated X‐PDT. b) In vivo real‐time fluorescence imaging of Hela tumor‐bearing mice after iv injection of TBDCR NPs. c) The tumor growth of mice after receiving different treatments. d) Body weights of the mice after different treatments. e) Images of representative tumors taken from mice in different groups. f) H&E staining of tumor tissues of mice at end of measurement. Data presented means ± SD. n = 4, ***p < 0.001. Scale bar = 100 µm.

The excellent cancer cell ablation effect of **TBDCR NPs** in vitro prompted us to further investigate their imaging and X‐PDT therapeutic performance in vivo. As shown in **Figure** **5**a, the Hela/HelaR subcutaneous tumor models were established by inoculating the tumor cells on the flank region of the right hind leg of female athymic BALB/c nude mice. The tumor enrichment of **TBDCR NPs** in vivo was firstly investigated. **TBDCR NPs** were injected intravenously into mice via the tail vein. As illustrated in Figure 5b; Figure S38 (Supporting Information), the tumor area started to show observable fluorescence at 3 h post injection, which gradually increased and reached the maximum at 24 h post injection, implying that **TBDCR NPs** gradually enriched at the tumor region, which should be attributed to the EPR effect of their nanoscale sizes. Furthermore, the significant fluorescent signal of the tumor region could still be distinctly observed at 48 h, which adequately demonstrated that **TBDCR NPs** had a remarkable tumor retention effect. Ex vivo fluorescence images of various organs and tumor tissue from the mice were obtained after the mice were sacrificed at 48 h post injection (Figure S39, Supporting Information). The heart, lung, liver, spleen as well as kidney did not exhibit significant red fluorescent signal, nevertheless, the tumor sites continued to exhibit a large amount of red fluorescent signal, further indicating the excellent accumulation of **TBDCR NPs** at tumor sites.

After evaluating the distribution of **TBDCR NPs** in vivo through fluorescence imaging analysis, the Hela/HelaR‐skin‐tumor‐bearing female athymic BALB/c nude mice model was established by subcutaneous injection human cervical cancer cell line (HeLa) or radiation‐resistant Hela cells (HelaR) to the right hind leg of mice to assess the tumoricidal activity of **TBDCR NPs**. When the volume of tumors reached ≈80 mm^3^ (at day 14), the mice were randomly divided into eight groups (n = 4 mice per group): 1) HeLa + Control; 2) HeLa + **TBDCR NPs**; 3) HeLaR + Control; 4) HeLaR + **TBDCR NPs**; 5) HeLa + Control + X‐ray; 6) HeLa + **TBDCR NPs** + X‐ray; 7) HeLaR + Control + X‐ray; 8) HeLaR + **TBDCR NPs** + X‐ray. Among them, **TBDCR NPs** or PBS (Control) was intravenously injected into mice at day 6. The mice in groups 5–8 were irradiated with X‐rays at day 7 and day 9 (total X‐ray dosage of 16 Gy). Tumor volumes and body weights were monitored twice per week and the results were displayed in Figure 5b,c. It revealed that in the absence of X‐ray irradiation, the tumors from groups 1–4 showed rapid growth, and **TBDCR NPs** themselves exhibited negligible suppressive effect to HeLa and HeLaR tumors, and the tumors grow to 922.0 mm^3^ and 1104.8 mm^3^, respectively. Furthermore, only X‐ray irradiation (HeLa + Control + X‐ray and HeLaR + Control + X‐ray groups) exhibited partially inhibition of tumor growth in compared with tumors treated with PBS (as control) and **TBDCR NPs** only, indicating the limited antitumor efficiency of radiotherapy. Additionally, under the same dosages of X‐ray irradiation, the tumor growth in group HeLaR + X‐ray was significantly faster in mice than group HeLa + X‐ray, which confirmed that HelaR has greater resistance to radiation than Hela. In contrast, the tumor tissue growth was significantly inhibited in the presence of X‐ray and **TBDCR NPs**, where negligible tumor growth was observed for HeLa + **TBDCR NPs** + X‐ray group, with the final tumor volume was reduced to 66.5 mm^3^. While the tumors in group HeLaR + **TBDCR NPs** + X‐ray only increased to 152.0 mm^3^, ≈24.4% and ≈13.3% of those in groups HeLaR + Control and HeLaR + Control + X‐ray, respectively, indicating that **TBDCR NPs** mediated X‐PDT could significantly promote the antitumor effect of RT. Moreover, the tumoricidal activity effect of the **TBDCR NPs** under X‐ray irradiation was validated by the appropriate tumor photographs (Figure 5e). The mice from each group were sacrificed after treatment, and the tumor tissues were then collected for H&E staining to compare the tumor therapeutic effects of these presented groups. As indicated in Figure 5f; Figure S40 (Supporting Information), considerable cellular necrosis was seen in the tumors after **TBDCR NPs** and X‐ray irradiation in contrast to tumor cells in the other groups. These results indicated excellent in vivo X‐PDT performance of **TBDCR NPs**.

**Figure 6:**
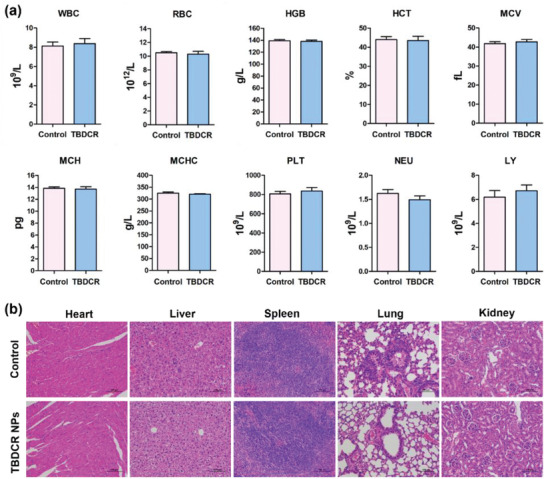
In vivo biosafety evaluation of TBDCR NPs. a) Routine blood indexes of mice after different treatments. b) H&E staining of the sectioned tissues (heart, liver, spleen, lung, and kidney) from mice intravenously injected with PBS or TBDCR NPs. Scale bar = 200 µm. Data presented means ± SD. n = 3.

The in vivo biosafety and biocompatibility of **TBDCR NPs** for conceivable biological and biomedical applications were further evaluated by the routine blood analysis and H&E staining of the main organs of mice. Initially, BALB/c nude mice were intravenously with **TBDCR NPs** by the tail vein and the control groups received the saline injection. At day 14 post injection, the blood samples were collected for examination. As shown in **Figure** **6**a, the blood levels including white blood cell (WBC, an indicator of inflammation), red blood cell (RBC, an indicator of anemia), and mean corpuscular volume (MCV, an indicator of changes in red blood cell volume), etc. showed hardly significant difference between **TBDCR NPs** and the control groups. Additionally, as shown in Figure 6b, the H&E experimental findings of the major organs (heart, liver, spleen, lung, and kidneys) showed no aberrant cell morphology or tissue abnormalities. All of these results demonstrated **TBDCR NPs** was biocompatible and appropriate for in vivo system.

## 3 Conclusion

In summary, we rationally designed and developed a novel pure organic AIE nanoscintillator for X‐ray induced type I and type II PDT. The introduction of heteroatoms (oxygen, nitrogen, and sulfur atoms) to **TBDCR** helps to improve X‐ray harvesting, which also promotes n‐π* conversion, speeds up the ISC process as well as enhances the triplet state exciton formation, strengthening ROS generation ability under X‐ray irradiation. Notably, with the help of AIE moiety, **TBDCR** showed aggregation‐enhanced emission, triplet exciton generation and ROS generation, and its ROS generation was greatly outperformed over commonly used PSs such as Ce6 or CV. Intriguingly, the formation of PEG crystalline shell leads to denser and more compact **TBDCR** packing in the intraparticle microenvironment, resulting in further improved ROS generation for **TBDCR NPs**. As a consequence, the pure organic nanoscintillator **TBDCR NPs** demonstrated excellent fluorescence as well as ^1^O_2_ and HO^•−^ generation under X‐ray irradiation without high‐Z inorganic element as energy transducers. Both in vitro and in vivo experimental results demonstrated the excellent X‐PDT antitumor performance of **TBDCR NPs** towards both HeLa tumors as well as radiation‐resistant HeLaR tumors. To the best of our knowledge, our **TBDCR NPs** represent the first work of pure organic scintillator for type I X‐PDT. Our proposed strategy shall pave new insight for the development of pure organic scintillators with excellent X‐ray harvesting and predominant free radical generation in aggregates, and we anticipate that our work could inspire more straightforward and effective type I organic scintillators for the clinical treatment of solid tumors via X‐PDT.

## Conflict of Interest

The authors declare no conflict of interest.

## Supporting information

Supporting Information

## Data Availability

The data that support the findings of this study are available in the supplementary material of this article.
