# Supplementary material for: Pure Organic AIE Nanoscintillator for X‐ray Mediated Type I and Type II Photodynamic Therapy
Source: Adv Sci (Weinh). 2023 Jul 9;10(26):2302395. doi: 10.1002/advs.202302395 (PMC10502865; doi:10.1002/advs.202302395)
Supplement: Supplementary file 1 — Supporting Information [file ADVS-10-2302395-s001.pdf]

ADVANCED SCIENCE
Open Access

Supporting Information

for *Adv. Sci.*, DOI 10.1002/advs.202302395

Pure Organic AIE Nanoscintillator for X-ray Mediated Type I and Type II Photodynamic Therapy

Yuewen Yu, Lisha Xiang, Xuanwei Zhang, Le Zhang, Zhiqiang Ni, Zhong-Hong Zhu, Yubo Liu, Jie Lan, Wei Liu, Ganfeng Xie, Guangxue Feng* and Ben Zhong Tang**

Supporting Information

Pure Organic AIE Nanoscintillator for X-ray Mediated Type I and Type II Photodynamic Therapy

Yuewen Yu, Lisha Xiang, Xuanwei Zhang, Le Zhang, Zhiqiang Ni, Zhong-Hong Zhu, Yubo Liu, Jie Lan, Wei Liu, Ganfeng Xie,* Guangxue Feng,* Ben Zhong Tang*

Y. Yu,[†] L. Zhang, Z. Ni, Z.-H. Zhu, Y. Liu, Prof. G. Feng*
State Key Laboratory of Luminescent Materials and Devices, Guangdong Provincial Key Laboratory of Luminescence from Molecular Aggregates, School of Materials Science and Engineering, AIE Institute, South China University of Technology, Guangzhou, 510640, China
E-mail: fenggx@scut.edu.cn (G. Feng)

Dr. L. Xiang,[†] X. Zhang, J. Lan
Division of Thoracic Tumor Multimodality Treatment and Department of Medical Oncology, Department of Radiation Oncology, Cancer Center, State Key Laboratory of Biotherapy, West China Hospital, Sichuan University, Chengdu, Sichuan 610041, China

Dr. W. Liu
Analysis and Testing Research Center, East China University of Technology, Nanchang 330013, China
State Key Laboratory of Chemo/Biosensing and Chemometrics, Hunan University, Changsha 410082, China
Prof. G. Xie*
Department of Oncology and Southwest Cancer Centre, Radiation Treatment Centre, Southwest Hospital, Third Military Medical University (Army Medical University), Chongqing 400038, China
E-mail: xiegf@aliyun.com (G. Xie);

Prof. B. Z. Tang*
School of Science and Engineering, Shenzhen Institute of Aggregate Science and Technology, The Chinese University of Hong Kong, Shenzhen, Guangdong 518172, China.
E-mail: tangbenz@cuhk.edu.cn (B. Z. Tang)

Y. Yu and L. Xiang contributed equally to this work.

Experiment procedures

1. Materials

All the solvents and reagents used in this work were of analytical grade. Malononitrile, phenyl isothiocyanate, bromoacetyl bromide, 4-bromobenzaldehyde and 3-(4, 5-dimethylthiazol-2-yl)-2, 5-diphenyltetrazolium bromide (MTT) were purchased from Energy Chemical Co., Ltd. 4-(Diphenylamino)phenylboronic acid was obtained from Soochiral Chemical Science & Technology Co., Ltd. The ROS indicators of dichlorodihydrofluorescein diacetate (DCFH-DA), dihydrorhodamine 123 (DHR123), and 9',10'-anthracenediyl-bis(methylene)-dimalonic acid (ABDA) was offered from Aladdin Co., Ltd. The intracellular ROS probe 2′,7′-dichlorodihydro fluorescein diacetate (DCFH-DA), and the DNA damage probe γ-H2AX were purchased from Beyotime biotechnology Co., Ltd. 2-[6-(4’-Hydroxy) phenoxy-3H-xanthen-3-on-9-yl] benzoate (hydroxyphenyl fluorescein, HPF) was purchased from AAT Bioquest, USA. DSPE-PEG_{2000} was offered from RuixiBiotech Co., Ltd. Human cervical cancer cell line HeLa were obtained from the ATCC (Manassas, VA, USA).

2. Instruments

^{1}H NMR and ^{13}C NMR spectra were detected on a Bruker AV 500 spectrometer. Shimadzu UV-2600 spectrophotometer was used to measure the UV-vis absorption spectrum. Steady photoluminescence (PL) spectra and delayed emission spectra were conducted on a Horiba Fluoromax-4 spectrofluorometer. The lifetime was obtained on Edinburgh FLS1000. Radioluminescence (RL) spectra were obtained employing a D8 Focus diffractometer (Bruker) with Cu Kα radiation (λ = 0.15405 nm) at 30 KeV and a FLAME-S-XR1-ES spectrophotometer (Ocean Optics USB2000 + XR1-ES). Powder X-ray diffraction (PXRD) data were measured by Smartlab SE (Rigaku Corporation). Particle size and Zeta potential analysis were performed on a Malvern Zetasizer Nano-S90. TEM images were obtained on JEM 2100F. High resolution mass spectrometric (HRMS) data were carried out using LTQ Orbit rap XL instruments. Photocurrent experiment was carried out by CHI 852C. Confocal laser

scanning microscopy (CLSM) images were obtained on a Zeiss LSM7 DUO Laser Scanning Confocal Microscope and Leica TSC-SP5, Germany. X-ray radiation was performed by a 6MV-X linear accelerator (Varian, 23-EX). Small animals' fluorescence imaging was carried out by Fusion FX6.EDGE living imaging system.

3. Synthesis

3.1. Synthesis of compound Rho

Malononitrile (2.2 g, 33 mmol) was dissolved in dioxane (20 mL) and cooled to 0 □, subsequently, the above mixture was added dropwise to a stirred mixture of phenylisothiocyanate (4.46 g, 33 mmol) and dry KOH powder (1.85g, 33 mmol) in dioxane (50 mL), which was previously cooled to 0 □. The temperature of the stirred mixture was kept at 0-5 □ during malononitrile addition. Filtered off the precipitated and washed with cold dioxane for 2 times, further dried it in vacuum oven. Next, a solution of the salt (4.78 g, 20 mmol) in distilled water (20 mL), and bromoacetyl bromide in ethanol was added, the reaction combination was stirred at room temperature for approximately two hours. Then, filtered off the precipitated, dried as well as crystallized it from ethanol to obtain the pure compound **Rho** with the yield of 85%. ^{1}H NMR (400 MHz, DMSO-d_6) δ 7.59-7.51 (m, 3H), 7.46 (dd, J = 8.0, 1.4 Hz, 2H), 4.34 (s, 2H). ^{13}C NMR (101 MHz, DMSO-d_6) δ 175.31, 173.01, 133.81, 131.57, 130.04, 129.59, 114.76, 110.43, 54.03, 34.52.

3.2. Synthesis of compound 1

4-(Diphenylamino)phenylboronic acid (0.5 g, 1.72 mmol), 4-bromo-2-benzaldehyde (0.35 g, 1.72 mmol) and tetrakis (triphenylphosphine)palladium (0) (35 mg, 30 μmol) were dissolved in THF (10 mL) in two-neck round-bottom flask. 1 mL saturated potassium carbonate solution (2 M) was then added to the reaction mixture under stirring. The reaction was refluxed at 80 °C with N_2 atmosphere for overnight. The solvent was removed under low pressure after the combination had cooled. The residue was dissolved in DCM (50 mL) and then extracted with brine and water (3×50 mL) for three times. The organic layer was separated and dried over anhydrous

Na_2SO_4. The residue was purified using flash column chromatography on silica gel (petroleum ether: ethyl acetate, 50:1, v/v) to get the compound **1** as yellow solid with the yield of 83%. ^{1}H NMR (500 MHz, $CDCl_3$) δ 10.03 (s, 1H), 7.92 (d, *J* = 8.2 Hz, 2H), 7.72 (d, *J* = 8.2 Hz, 2H), 7.52 (d, *J* = 8.6 Hz, 2H), 7.29 (t, *J* = 7.8 Hz, 4H), 7.14 (dd, *J* = 8.5, 2.0 Hz, 6H), 7.07 (t, *J* = 7.3 Hz, 2H). ^{13}C NMR (126 MHz, $CDCl_3$) δ 191.87, 148.42, 147.32, 146.62, 134.67, 132.78, 130.34, 129.40, 128.02, 126.89, 124.88, 123.47, 123.11.

3.3. Synthesis of compound TBDCR

Compound **1** (0.18 g, 0.5 mmol), **Rho** (0.12 g, 0.5 mmol) as well as ammonium acetate (0.050 g, 0.64 mmol) was dissolved in 15 mL acetic acid, and refluxed at 120 °C for 12 h under Argon. After cooling to 25 °C, the reaction was quenched by water (100 mL). Then, the filtered off the precipitate and washed with water (100 mL). The bulk product was further purified by flash column chromatograph (silica gel, dichloromethane/EtOH = 20/1) as the eluent to get the product as red solid with the yield of 85%. ^{1}H NMR (400 MHz, $CDCl_3$) δ 8.02 (s, 1H), 7.76 (d, *J* = 8.3 Hz, 2H), 7.63 (dd, *J* = 7.6, 6.0 Hz, 4H), 7.54 (d, *J* = 8.6 Hz, 2H), 7.34 (dd, *J* = 7.0, 5.9 Hz, 3H), 7.29 (d, *J* = 7.5 Hz, 3H), 7.16 (dd, *J* = 8.5, 2.1 Hz, 6H), 7.08 (t, *J* = 7.3 Hz, 2H). ^{13}C NMR (101 MHz, $CDCl_3$) δ 166.00, 165.63, 148.58, 147.27, 144.08, 136.98, 132.69, 132.19, 131.88, 131.49, 130.46, 130.27, 129.46, 128.75, 127.85, 127.38, 125.02, 123.63, 122.99, 115.52, 113.04, 109.31, 58.08. HRMS (ESI): m/z calcd. for $C_{37}H_{24}N_4OSNa^+$ = 595.1563, found m/z = 595.1567.

4. Preparation of TBDCR Nanoparticles

Nanoparticles were formulated by a nanoprecipitation approach using an amphiphilic co-polymer, DSPE-PEG_{2000}. In brief, **TBDCR** (1 mg), DSPE-PEG_{2000} (3 mg) were dissolved in THF (1 mL), which was then added into Milli-Q water (10 mL) and followed by 2 min ultrasound sonication. The mixture was further stirred at 600 rpm in the dark in a fume hood overnight for THF evaporation to achieve the aqueous solution. The obtained nanoparticles were stored at 4 °C for further use.

5. ROS Generating Ability Test

5.1. General ROS detection

5.1.1 General ROS detection under white light irradiation

DCFH was employed as a probe to examine the generation of general ROS (λ_{ex} = 480 nm, λ_{em} = 525 nm). 2,7-dichlorodihydrofluorescein diacetate (DCFH-DA, 0.5 mL, 1 mM in ethanol) and aqueous NaOH solution (2 mL, 1.0 mM) were combined to create DCFH, which was then processed for 30 min at ambient temperature. After neutralizing the hydrolysate with 7.5 mL of PBS buffer solution, a stock solution of DCFH with the concentration of 50 μM was obtained. Subsequently, PBS buffer solution containing DCFH (50 μM) was added with 10 μM **TBDCR** as well as other commercially available PSs (Ce6 and CV) (stock solution: 1 mM in DMSO). The PL spectrum of DCFH in the mixure was recorded under white light irradiation (50 $mWcm^{-2}$) in a cuvette. The rate of general ROS production was determined by measuring the fluorescence intensity at 525 nm.

5.1.2 General ROS detection under X-ray irradiation

DCFH-DA (0.5 mL, 1 mM in ethanol) and aqueous NaOH solution (2 mL, 1.0 mM) were combined to create DCFH, which was then processed for 30 min at ambient temperature to obtain the stock solution of 200 μM. 20 μL DCFH (stock solution) was added to different volume **TBDCR NPs** and other commercially available PSs (Ce6 and CV) PBS buffer solution, predesignated volumes of PBS were added to the mixture to achieve a final concentration of 10 μM for DCFH, and various concentrations of 0, 5, 10, 20, 40 μg/mL for **TBDCR NPs** as well as other commercially available PSs (Ce6 and CV), respectively. Then the PL intensity of DCFH was recorded through SpectraMax i3x (Molecular Devices) after X-ray irradiation (4 Gy) in 96-well plates. The rate of general ROS production was determined by measuring the fluorescence intensity at 525 nm. The concentration of stock solution of **TBDCR NPs** was 1 mg/mL in ultrapure water, and the concentration of stock solution of **Ce6**, and **CV** was 1 mg/mL in DMSO.

5.2. Hydroxyl radical ($OH^{\bullet-}$) detection

5.2.1 $OH^{\bullet-}$ detection under white light irradiation

Hydroxyphenyl fluorescein (HPF) was used as a probe to examine the generation of hydroxyl radical (λ_{ex} = 480 nm, λ_{em} = 514 nm). 10 μM **TBDCR** as well as other commercially available PSs (Ce6 and CV) (stock solution: 1 mM in DMSO) were respective dissolved in PBS buffer solution containing 10 μM HPF (stock solution: 5 mM in DMF). The PL spectrum of HPF that was mixed with various PSs was recorded under white light irradiation (50 $mWcm^{-2}$) in a cuvette. The rate of hydroxyl radical production was determined by measuring the fluorescence intensity at 514 nm.

5.2.2 $OH^{\bullet-}$ detection under X-ray irradiation

20 μL HPF (stock solution: 200 μM in DMF) was added to different volume **TBDCR NPs** and other commercially available PSs (Ce6 and CV) PBS buffer solution, and then added a certain volume of PBS to achieve final concentrations of 10 μM for HPF, and the concentration of **TBDCR NPs** as well as other commercially available PSs (Ce6 and CV) were 0, 5, 10, 20, 40 μg/mL, respectively. Then the PL intensity of HPF was recorded through SpectraMax i3x (Molecular Devices) after X-ray irradiation (4 Gy) in 96-well plates. The rate of hydroxyl radical production was determined by measuring the fluorescence intensity at 514 nm. The concentration of stock solution of **TBDCR NPs** was 1 mg/mL in ultrapure water, and the concentration of stock solution of **Ce6** as well as **CV** was 1 mg/mL in DMSO.

5.3. Singlet oxygen ($^1O_2$) detection

5.3.1 $^1O_2$ detection under white light irradiation

9,10-anthracenediyl-bis(methylene)-dimalonic acid (ABDA) was used as indicator for detection of $^1O_2$ in PBS buffer solution. 10 μM **TBDCR** as well as other commercially available PSs (Ce6 and CV) (stock solution: 1 mM in DMSO) were respective dissolved in PBS buffer solution containing 50 μM of ABDA (stock solution: 10 mM in DMSO). After irradiating the mixture by white light irradiation (50 $mWcm^{-2}$) in a cuvette, subsequently, the absorption spectra were monitored. The

reduction in absorbance compared to the beginning level was measured at 378 nm to determine the rate of decomposition of ABDA ($^1O_2$ generation rate).

5.3.2. $^1O_2$ detection under X-ray irradiation

20 μL ABDA (stock solution: 1 mM in DMSO) was added to different volume **TBDCR NPs** aqueous solution, and then added a certain volume of PBS to achieve final concentrations of 50 μM for ABDA, and the concentration of **TBDCR NPs** were 0, 5, 10, 20, 40 μg/mL, respectively. Then the absorption intensity of ABDA was recorded through SpectraMax i3x (Molecular Devices) after X-ray irradiation (4 Gy) in 96-well plates. The absorption intensity at 378 nm was measured to determine the $^1O_2$ generation rate. The concentration of stock solution of **TBDCR NPs** was 1 mg/mL in ultrapure water, and the concentration of stock solution of **Ce6** as well as **CV** was 1 mg/mL in DMSO.

6. Electron Paramagnetic Resonance (EPR) for detecting ROS

The electron paramagnetic resonance (EPR, Bruker A300) spectra were employed to evaluate the generation capability of ROS. DMPO and TEMP were employed as trapping agents for $HO^{\bullet-}$ and $^1O_2$, respectively. 100 μM **TBDCR** (stock solution: 1 mM in CH_3OH) was dissolved in PBS buffer solution that included 50 μM of each of the trapping agent (stock solution: 10 mM in CH_3OH), respectively. The EPR spectra of the mixtures were recorded before and after white light exposure (xenon lamp, 10 min, 100 mW cm^{-2}).

7. Photocurrent experiment

Photocurrent experiments were conducted by using aconventional three-electrode cell system as well as an electrochemical workstation (CHI 852c. chenhua, China). As the working electrode, the prepped electrode was used. The reference electrode was a saturated Ag/AgCl electrode, and the counter electrode was a platinum electrode. Unless otherwise specified, all potentials were referred to the saturated Ag/AgCl. As a source of visible light, MAX-302 xenon (Asahi Spectra) bulb with an ultraviolet cut-filter (> 420 nm) was employed. Linear sweep voltammetry (LSV) curves spanning

from -1.0 V to 1.0 V *vs* Ag/AgCl were measured at a scanning rate of 10 mV s^{-1}. Electrochemical impedance spectroscopy (EIS) was performed with a frequency range from 100 mHz to 100 kHz at a bias of + 0.6 V (vs Ag / AgCl) in 0.5 M Na_2SO_4 solution.

8. Cellular study

8.1. Cell cultures

HeLa cells were cultured in RPMI 1640 medium (GIBCO) supplemented with 10% fetal bovine serum (FBS) (GIBCO), penicillin (100 U/mL), and streptomycin (0.1 mg/mL. Cells were incubated in 5% CO_2 at 37 °C and passaged 2-3 times weekly.

8.2. Cellular uptake analysis

HeLa cells were grown at 37 °C in a confocal imaging plate. HeLa cells were initially incubated at 37 °C for various periods (2, 4, 6, 8 h) in 1 mL medium containing **TBDCR NPs** (10 μM). After the predetermined incubation period, the medium was removed, and the cells were washed three times with PBS before being stained for 30 min with medium containing Hoechest 33342 (100 nM, stock solution: 100 μM in DMSO). CLSM was used to photograph the cells after they had been washed twice with PBS. The excitation wavelength for **TBDCR NPs** was 405 nm, and the emission wavelength was 550-700 nm. The excitation wavelength was 405 nm as well as the emission wavelength was 430-470 nm for Hoechest 33342.

8.3. Confocal co-localization

HeLa cells were grown at 37 °C in a confocal imaging plate. HeLa cells were initially cultured for 6 hours at 37 °C in 1 mL medium containing **TBDCR NPs** (10 μM). The medium was then removed, and the cells were washed three times with PBS. The cells were then incubated for 30 min in medium containing BODIPY 493/501 (500 nM, standard solution: 100 μM in DMSO). CLSM was used to photograph the cells after they had been washed twice with PBS. The excitation wavelength for **TBDCR NPs** was 405 nm, and the emission wavelength was 550-700 nm. The excitation wavelength was 488 nm as well as the emission wavelength was 500-515 nm for

BODIPY 493/501.

8.4. Cell lines and preparation of radio-resistant HelaR cells

HeLa cells were cultured in RPMI 1640 medium (GIBCO) supplemented with 10% FBS (GIBCO), penicillin (100 U/mL), and streptomycin (0.1 mg/mL). Cells were passaged 1-2 times weekly while being grown at 37 °C in 5% CO_2. HeLa cells were grown in 75 cm^2 culture plates until they were about 75% confluent, at which point they were exposed to 2 Gy of X-ray radiation at room temperature using a high energy linear accelerator running at 6 MV. The growth medium was replaced right away following irradiation, and the cells were then put back into the incubator. HeLa cells were trypsinized, counted and passaged into fresh culture flasks after they had grown to a confluence of about 90%. When the cells had approximately 75% confluence, they underwent another 2 Gy treatment. The X-ray resistant subline (HeLaR), was created through constant sublethal radiation for six months at a dose of 2 Gy delivered 25 times for a total of 50 Gy. Without using ionizing radiation, the progenitor cell line (HeLa) was trypsinized, counted as well as passaged under the same circumstances. The radioresistant subline was cultured for more than 1-2 months following the final irradiation before being utilized in the analyses to account for acute effects of IR.

8.5. Cell viability test

Hela or HelaR cells were seeded per well in 96-well plates with a density of 2.5×10^4 cells/mL. The cells were incubated at 37 °C for 24 hours in the dark after being 80% confluent with various amounts of **TBDCR NPs** (0 μg/mL, 0.5 μg/mL, 1 μg/mL, 2 μg/mL, 5 μg/mL, 10 μg/mL, 20 μg/mL, 40 μg/mL and 50 μg/mL) suspended in cell culture medium. The PBS buffer was used to wash the experimental and control wells twice before adding newly prepared MTT solution (100 μL, 0.5 mg/mL). The MTT solution was meticulously removed from each well after 4 hours of incubation at 37 °C, and 100 μL of dimethyl sulfoxide was then added. After gently shaking the plate for 10 min, the absorbance of MTT at 570 nm was monitored by a microplate

reader (FLUOstar Omega) after gently shaking the plate for 10 minutes at room temperature to determine cell viability. For cellular toxicity with X-ray irradiation, 2.5×10^4 cells/mL cells seeded per well in 96-well plates. After 80% confluence, the cells were incubated with control or **TBDCR NPs** (50 μg/mL) for 24 h, subsequently, the cells were washed with fresh culture medium and then irradiated with X-ray under various dosages. Finally, MTT assay was conducted on cells after further 2 days incubation. The experimental and control wells were washed twice with PBS buffer and then added with freshly MTT solution (100 μL, 0.5 mg/mL). After incubation at 37 □ for 4 h, the MTT solution was removed carefully from each well and 100 μL of dimethyl sulfoxide was added. After gently shaking the plate for 10 min, the absorbance of MTT at 570 nm was monitored by a microplate reader (FLUOstar Omega) after gently shaking the plate for 10 minutes at room temperature to determine cell viability.

8.6. Measurement of intracellular ROS levels and DNA damage

Confocal fluorescence imaging and flow cytometry measurement were used to measure cellular ROS levels with of intracellular ROS probe DCFH-DA and HPF. Hela and HelaR cells were added per well in 96-well plates with a density of 2.5×10^4 cells/mL, respectively. About 3×10^5 cells were harvested, following incubation with 50 μg/mL **TBDCR NPs** for 24 h in the dark, DCFH-DA or HPF (10 μM) was loaded into the cells at 37 °C for 20 min, and the cells were washed twice with PBS and then exposed to X-ray (8 Gy) for irradiation. Then the cells were harvested, washed as well as resuspended in serum-free RPMI culture medium. DCFH-DA fluorescence (excitation wavelength 485 nm, and emission wavelength 525 nm) and HPF-fluorescence (excitation wavelength 490 nm, and emission wavelength 514 nm) were analyzed by flow cytometry, respectively. After accounting for autofluorescence, the mean fluorescence intensity (MFI) and fold change compared to the unirradiated control were calculated. For confocal imaging, the cell incubation process and the concentration of the reagents used as well as the X-ray dosage were same as flow cytometry. The fluorescence probe DCFH-DA or HPF and incubated at 37 °C for 20

min, or loaded with DNA Damage probe γ-H2AX and incubated at room temperature for 1 hour. Cells were carefully washed with PBS before imaging with a laser confocal scanning microscope.

9. TBDCR NPs-mediated X-PDT *in vivo*

9.1. Ethics statement

Experiments on animals were carried out with the approval of the Laboratory Animal Welfare and Ethics Committee of Army Medical University (Approval ID: AMUWEC20227013), in accordance with the State Science and Technology Commission Regulations for the Administration of Affairs Concerning Experimental Animals. (1988, China).

9.2. *In vivo* imaging

TBDCR NPs (1 mg/mL, 100 μL/mouse) was injected intravenously into mice *via* the tail vein. At predesignated time points, *in vivo* fluorescence imaging was visualized on Fusion FX6.EDGE Spectrum imaging system. The excitation was 405 nm, and the collected emission was above 650 nm.

9.3. *In vivo* tumor models

Female athymic BALB/c nude mice 4-6 weeks old were purchased from the Institute of Experimental Animal of Army Medical University (Chongqing, China). Mice were maintained under specific pathogen-free conditions. The mice were subcutaneously injected with HeLa or HeLaR cells (1×10^6 cells in 100 μL PBS/mouse) at the right hind limb (on day 0). BALB/c nude mice bearing xenografts (0.5-0.6 cm in diameter) were randomly divided into eight groups (n = 4 mice per group): HeLa + control; HeLa + **TBDCR NPs**; HeLaR + control; HeLaR + **TBDCR NPs**; HeLa + control + X-ray; HeLa + **TBDCR NPs** + X-ray; HeLaR + control + X-ray; HeLaR + **TBDCR NPs** + X-ray. When the volume of tumors reached ~80 mm^3 (at day 6), the mice were injected with control (PBS) or **TBDCR NPs** (1 mg/mL, 100 μL/mouse), then the mice were immobilized in a customized harness that exposed the right hind leg while shielding the remainder of the body behind a block of 3.5 cm thick lead. Lead

shielded mice received 16 Gy of X-ray radiation to the exposed tumor, divided into 2 fractions on day 7, 9. Tumor volume (mm^3) was calculated as $0.52 \times L \times W \times T$ (Length, width, and thickness). Tumor volumes and body weights were monitored twice per week. After 42 days, the animals were sacrificed, and tumors were excised and weighed. Xenografts were harvested to be subjected to H&E staining.

9.4. *In vivo* biosafety assessment

BALB/c mice (6 in total) were randomly divided into two groups (3 in each group) and intravenous injection of 1× PBS (100 μL) and **TBDCR NPs** (100 μL, 1 mg/mL). The blood routine levels, including white blood cell (WBC), red blood cell (RBC), hemoglobin (HGB), hematocrit (HCT), mean corpuscular volume (MCV), mean corpuscular hemoglobin (MCH), mean corpuscular hemoglobin concentration (MCHC), blood platelet (PLT), lymphocytes (Ly), and neutrophils (NEU) were measured by Automatic animal blood cell analyzer XN2000 (Sysmex Biotechnology Co., Ltd, Japan) at 14 days post injection. The organs were collected at the 14 days post injection for H&E staining.

10. Statistical analysis

GraphPad Prism Software Version 7.0 was used for statistical analysis. Statistical comparisons were made by unpaired Student's t-test (between two groups) and one way ANOVA (for multiple comparisons) with Tukey's post-hoc analysis. P value < 0.05 was considered statistically significant. Quantitative data were shown as mean ± standard deviation (SD).

11.Supplementary Figures and Tables

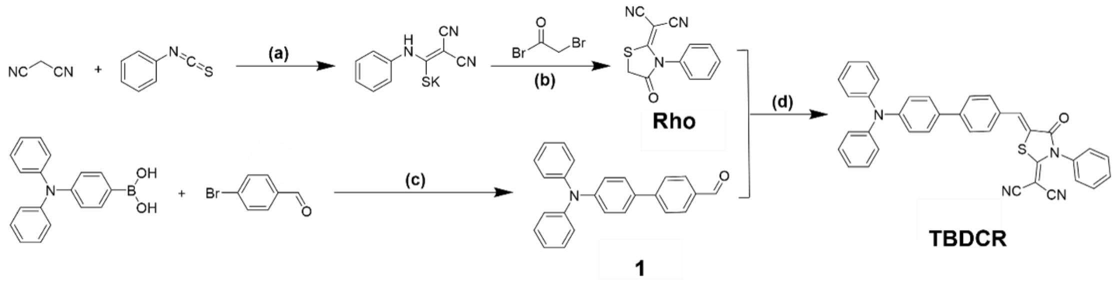

Scheme S1. Synthetic route of **TBDCR**. Reagents and conditions: (a) Malononitrile, dioxane, KOH, 0 □. (b) H_2O, ethanol. (c) Tetrakis(triphenylphosphine)palladium, K_2CO_3, THF/H_2O, 80 □. (d) Ammonium acetate, acetic acid, 118 □, overnight.

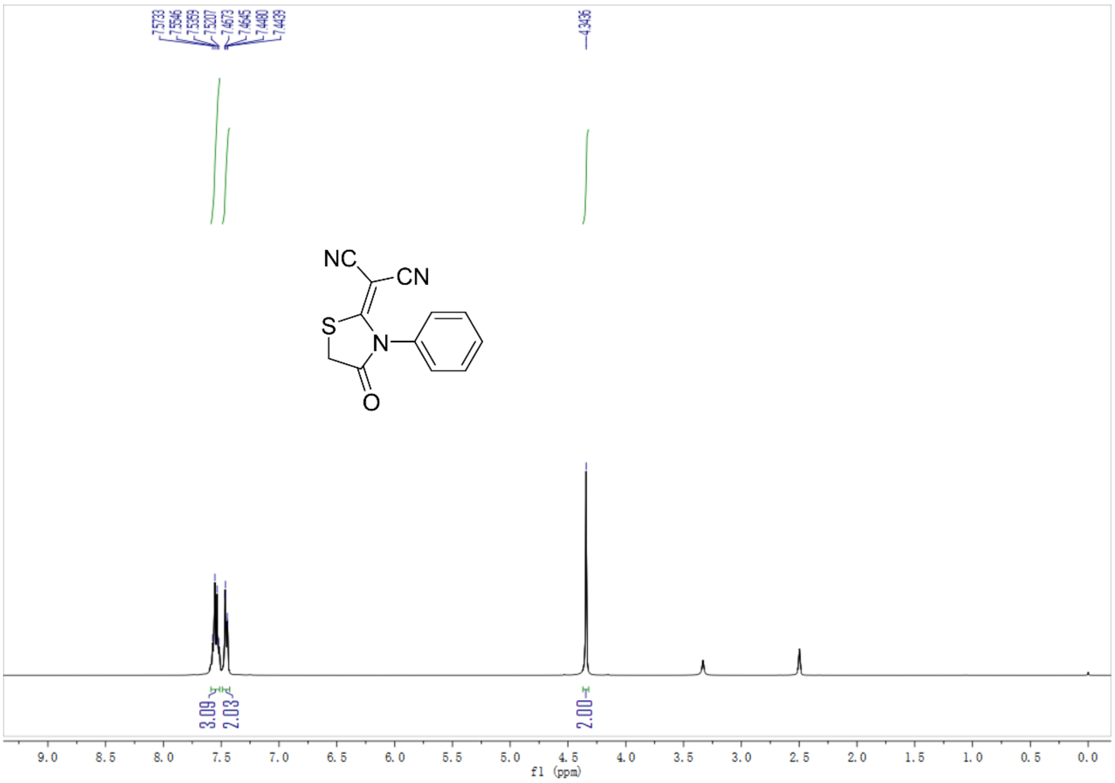

Figure S1. ^{1}H NMR spectrum of **Rho** in DMSO-d_6.

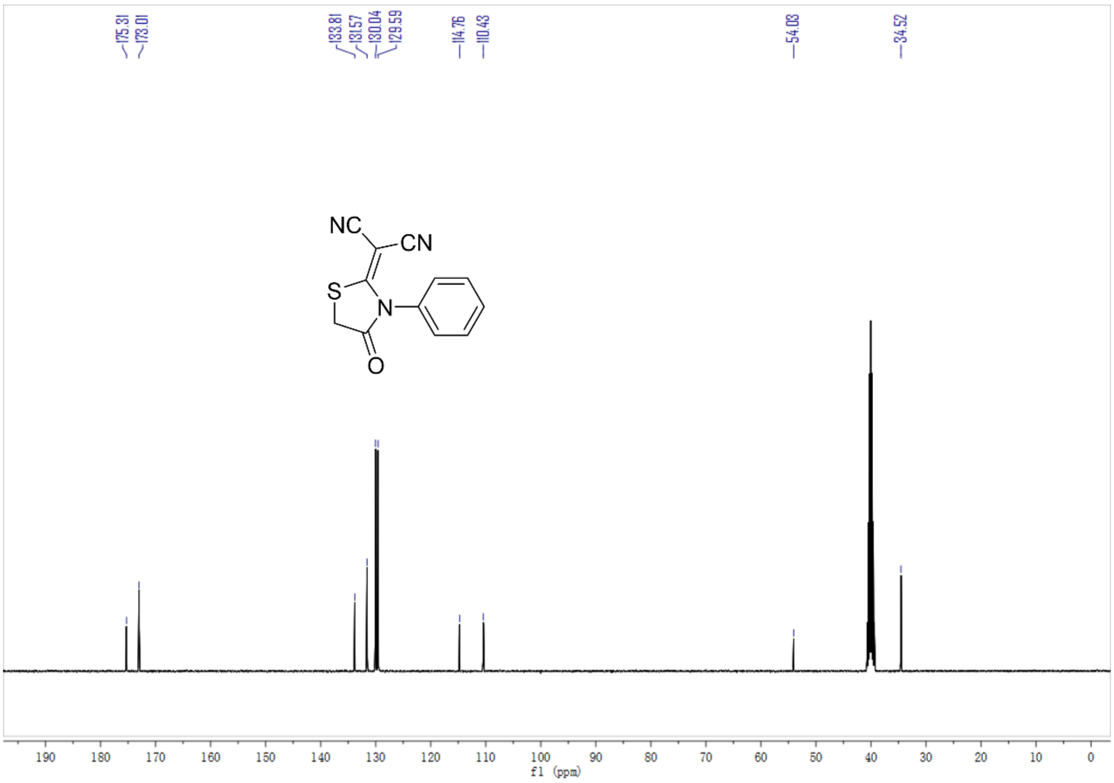

Figure S2. ^{13}C NMR spectrum of **Rho** in DMSO-d_6.

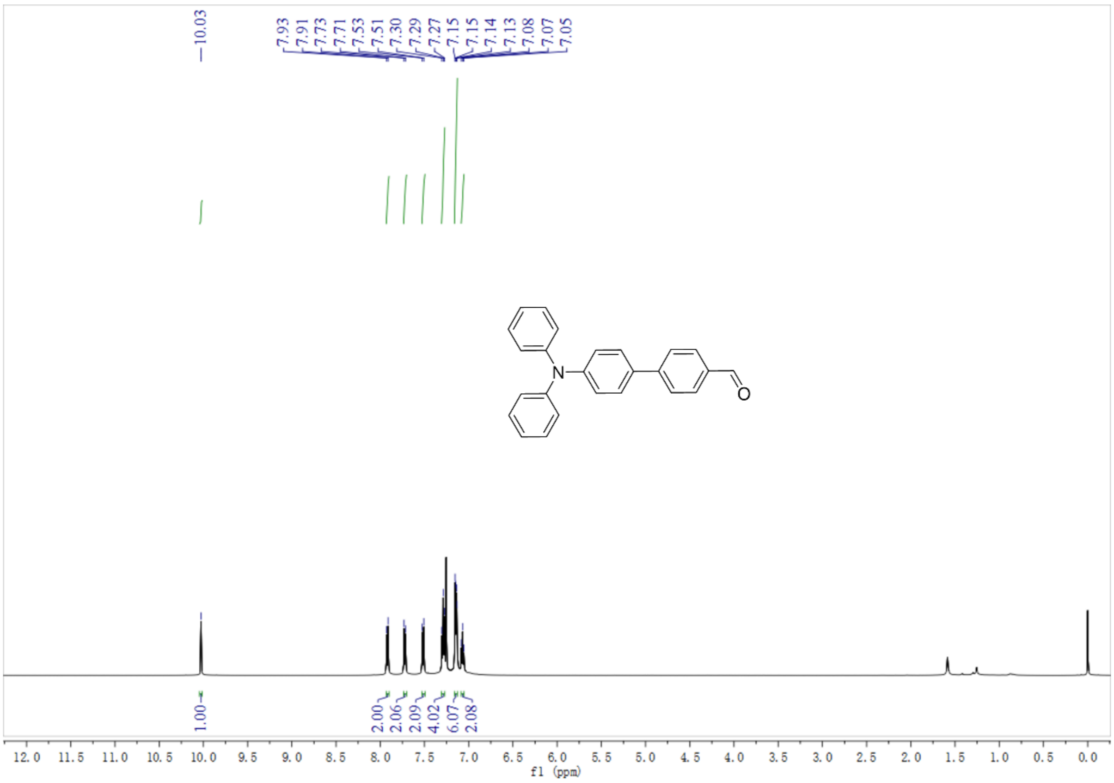

Figure S3. ^{1}H NMR spectrum of **1** in $CDCl_3$.

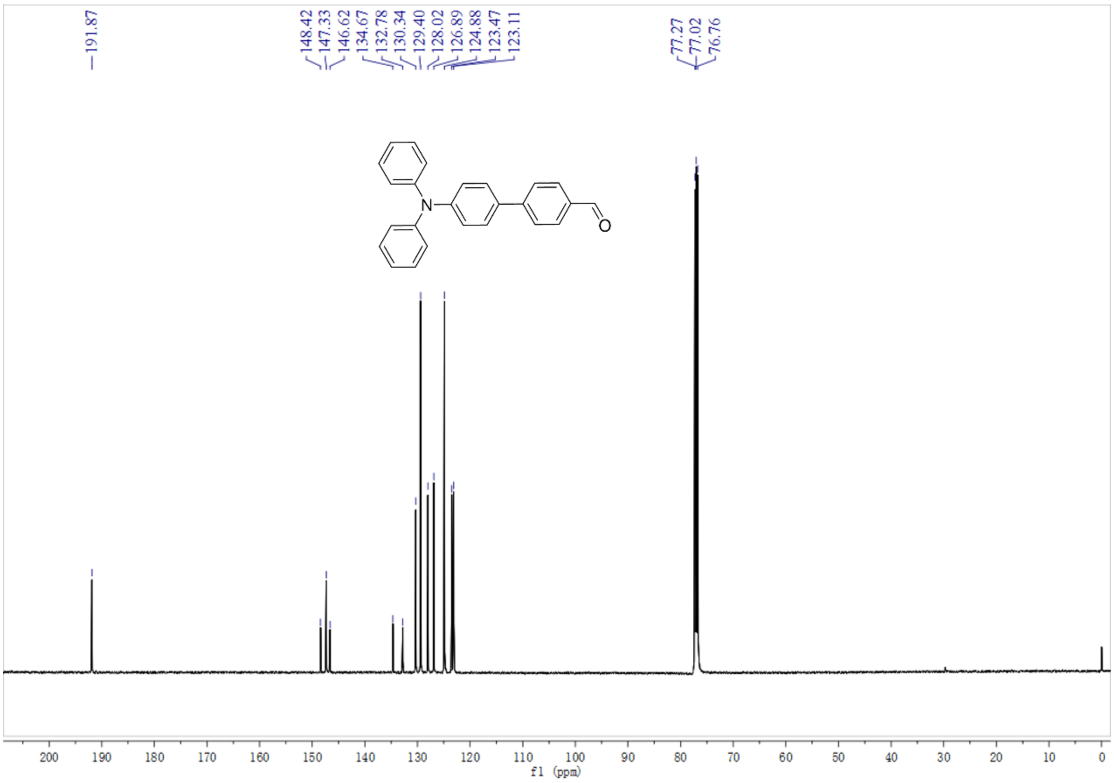

Figure S4. ^{13}C NMR spectrum of **1** in $CDCl_3$.

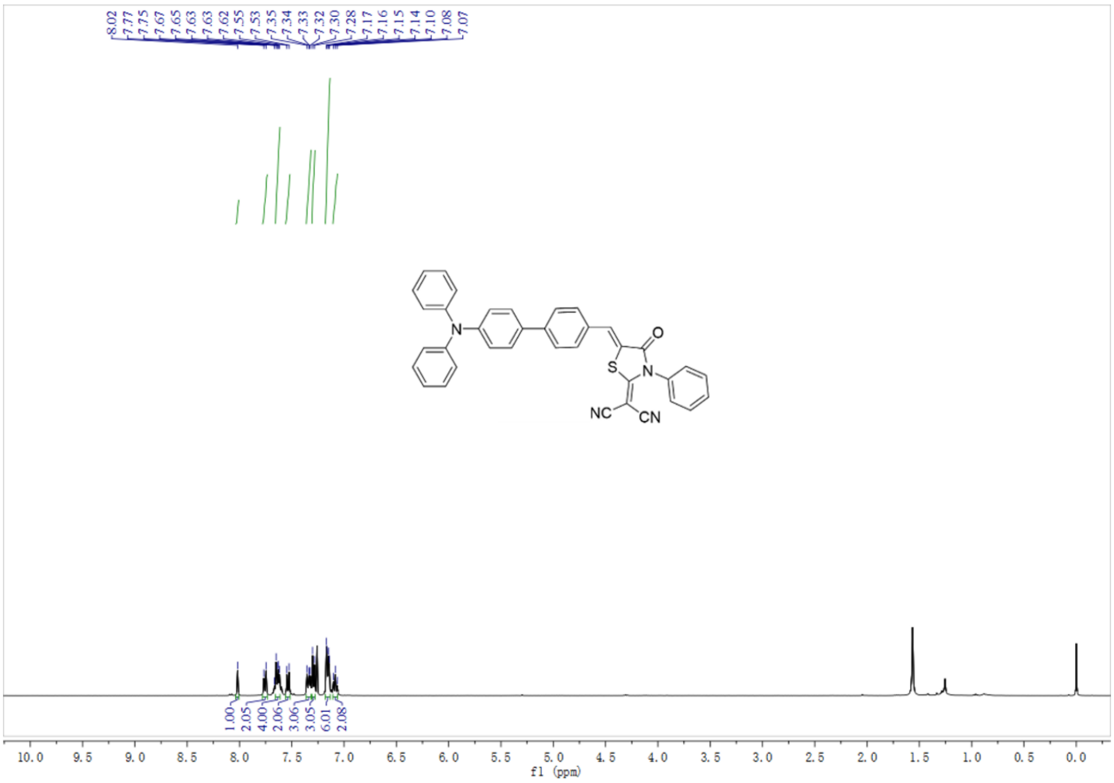

Figure S5. ^{1}H NMR spectrum of **TBDCR** in $CDCl_3$.

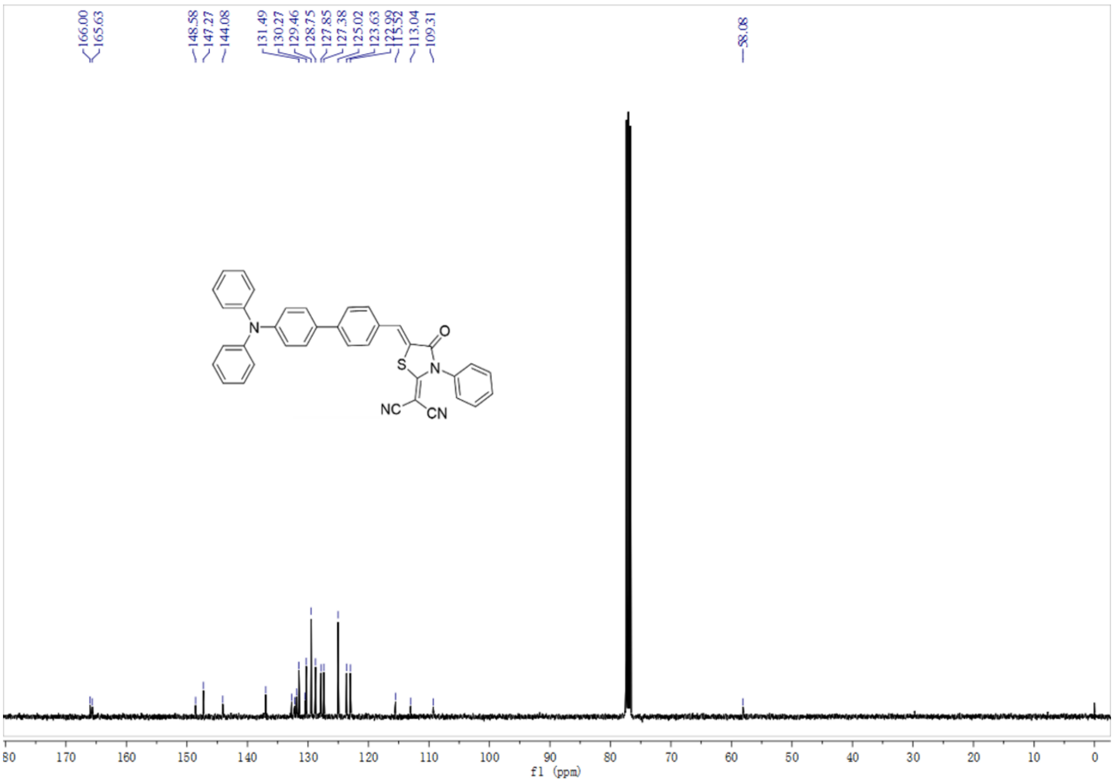

Figure S6. ^{13}C NMR spectrum of **TBDCR** in $CDCl_3$.

Generic Display Report

Analysis Info

Analysis Name	D:\Data\202209\62651\62651-2_P1-B-4_01_46521.d	Acquisition Date	9/26/2022 4:01:36 PM
Method	esi_pos_50-1000_with calibration_for 1min.m	Operator	HSJ
Sample Name	62651-2	Instrument	maXis impact
Comment			

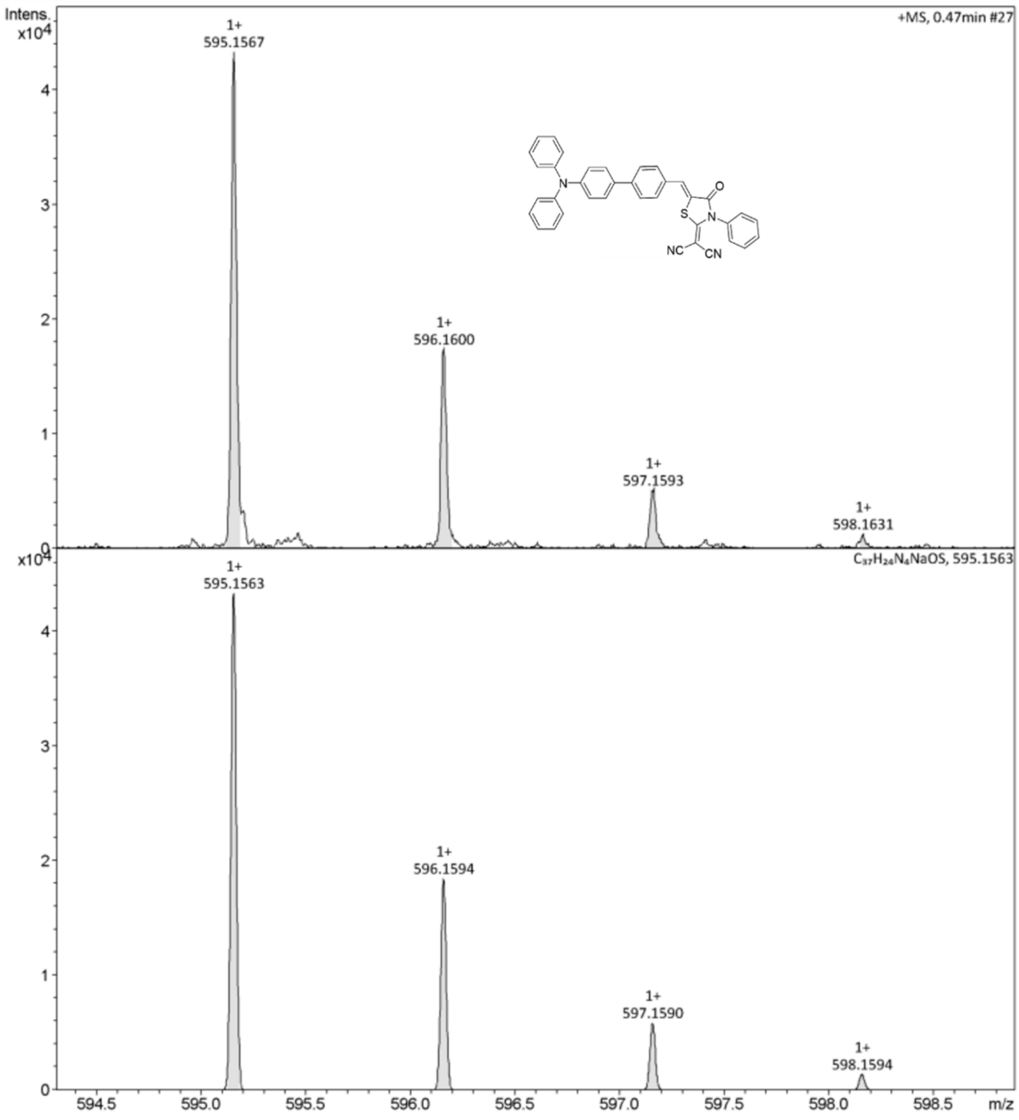

Bruker Compass DataAnalysis 4.1 printed: 9/26/2022 4:04:15 PM by: HSJ Page 1 of 1

Figure S7. HRMS spectrum of **TBDCR**.

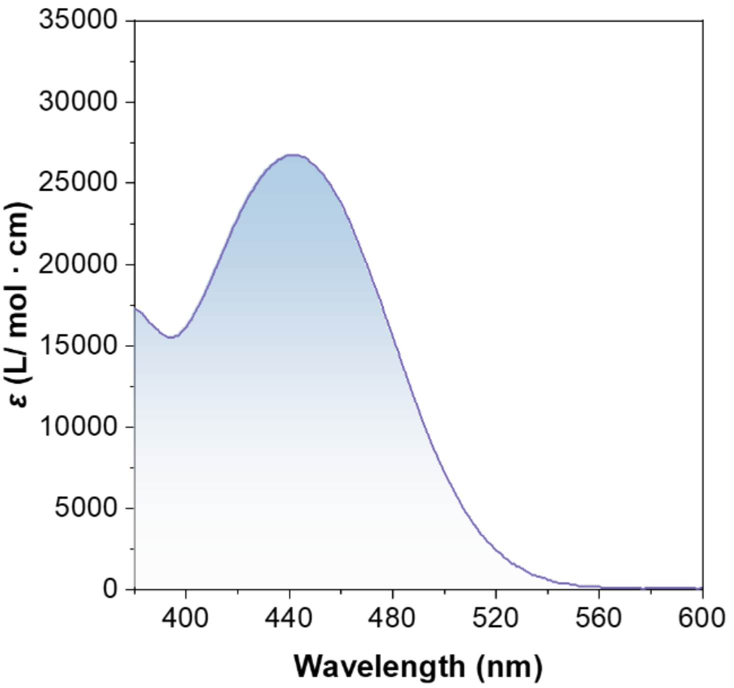

Figure S8. Absorption spectrum of **TBDCR** in THF solution (concentration: 10 μM).

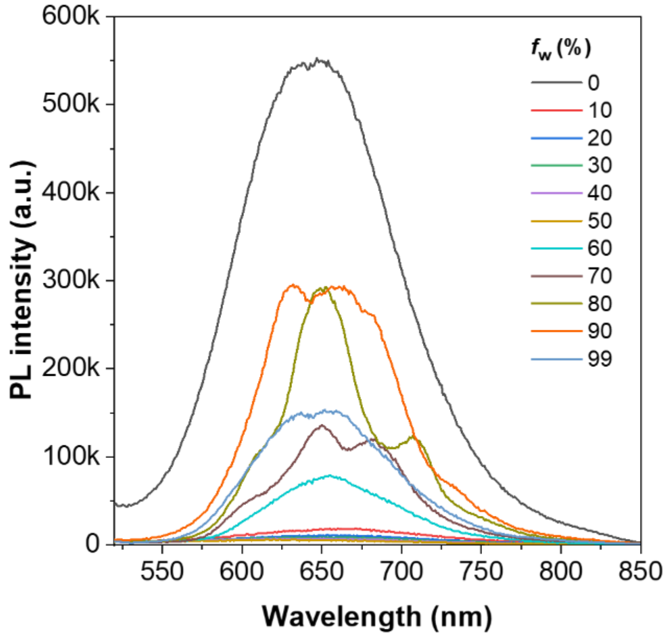

Figure S9. Plot of PL intensity versus water fraction of **TBDCR** in the THF/H_2O mixtures.

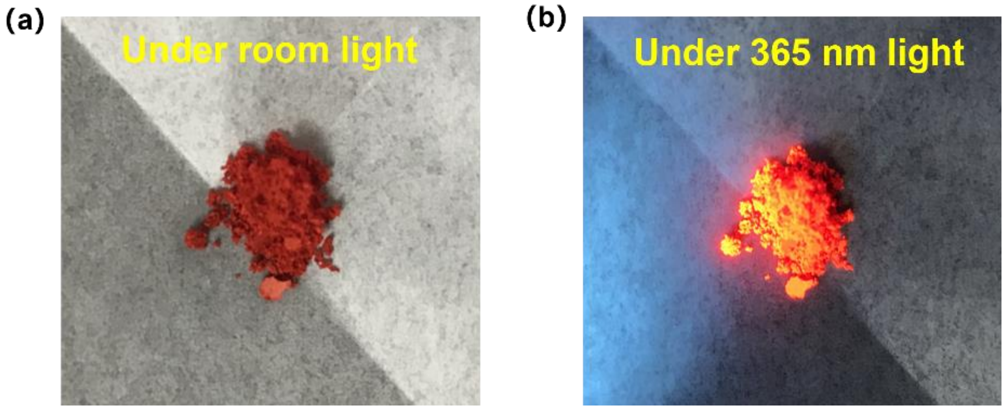

Figure S10. The photographs of **TBDCR** in solid state under room light and 365 nm light.

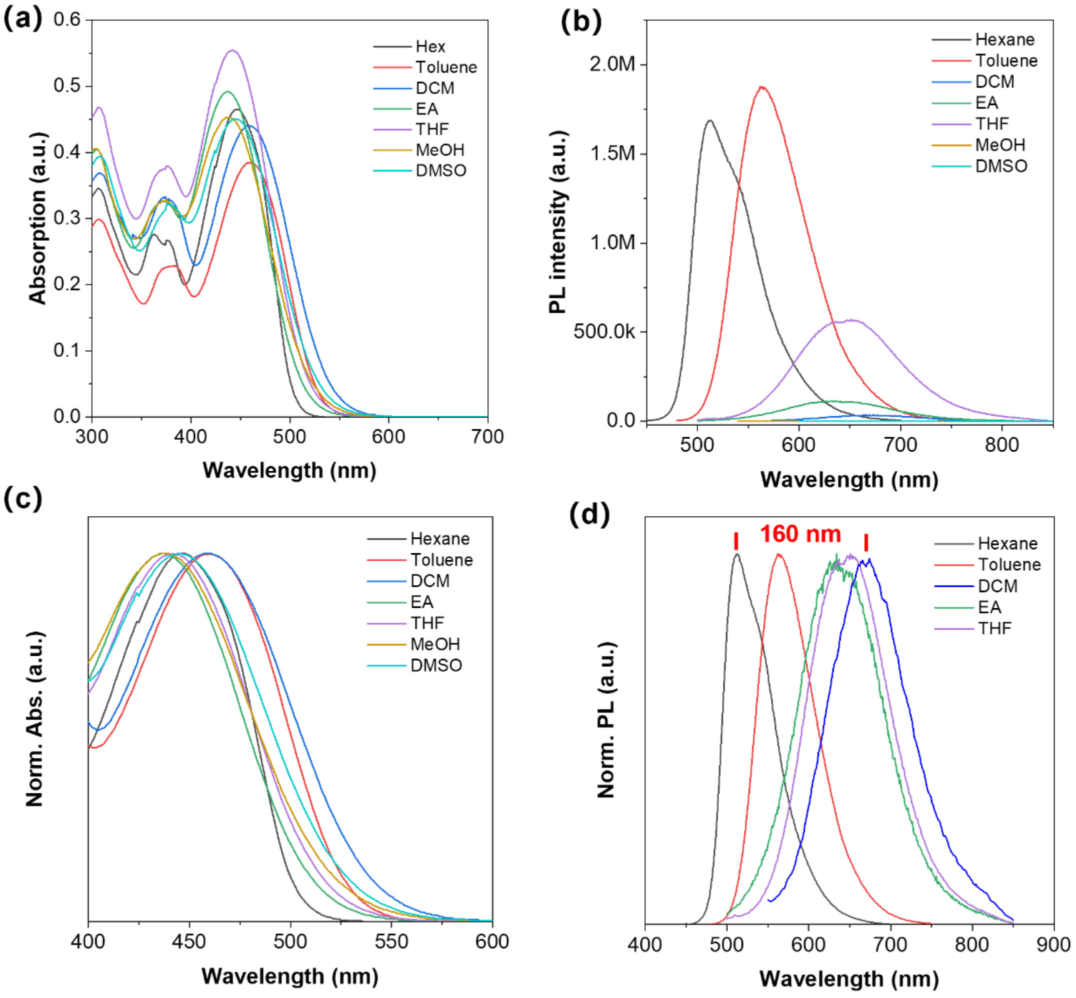

Figure S11. (a) Absorption and (b) PL spectra of **TBDCR** in different solvents, respectively. Normalized (c) absorption and (d) PL spectra of **TBDCR** in different solvents, respectively.

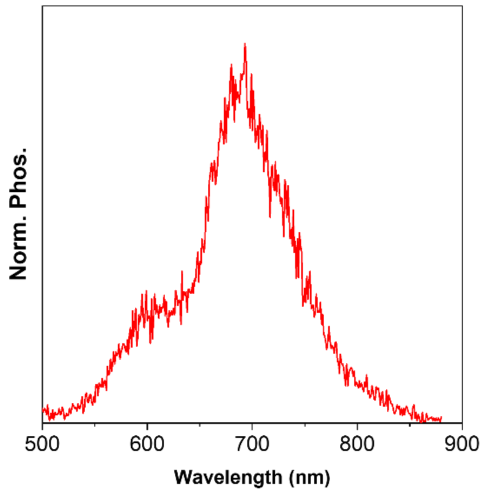

Figure S12. Time-gated phosphorescence (Phos.) spectrum of **TBDCR** in solid state.

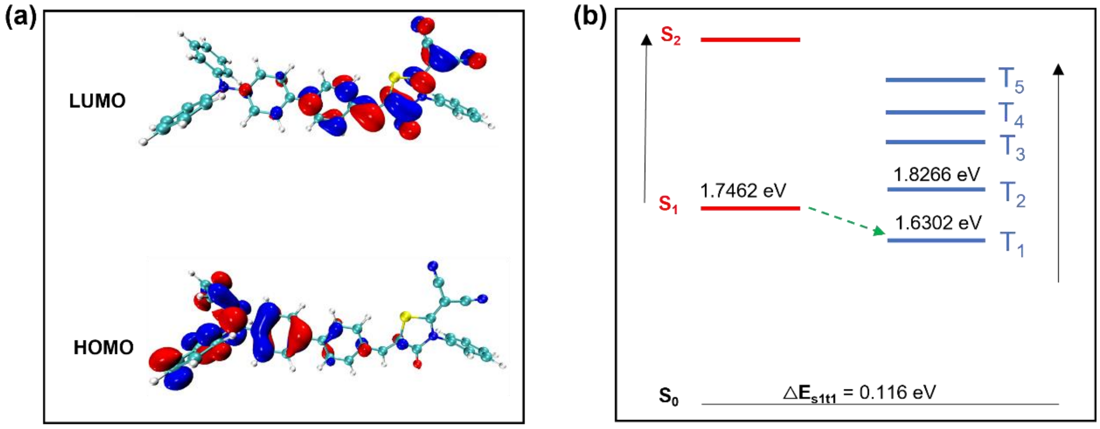

Figure S13. (a) HOMO and LUMO distribution, and (b) energy levels of excited singlet and triplet states of **TBDCR** calculated by TD-DFT (B3LYP/6-31G (d, p)).

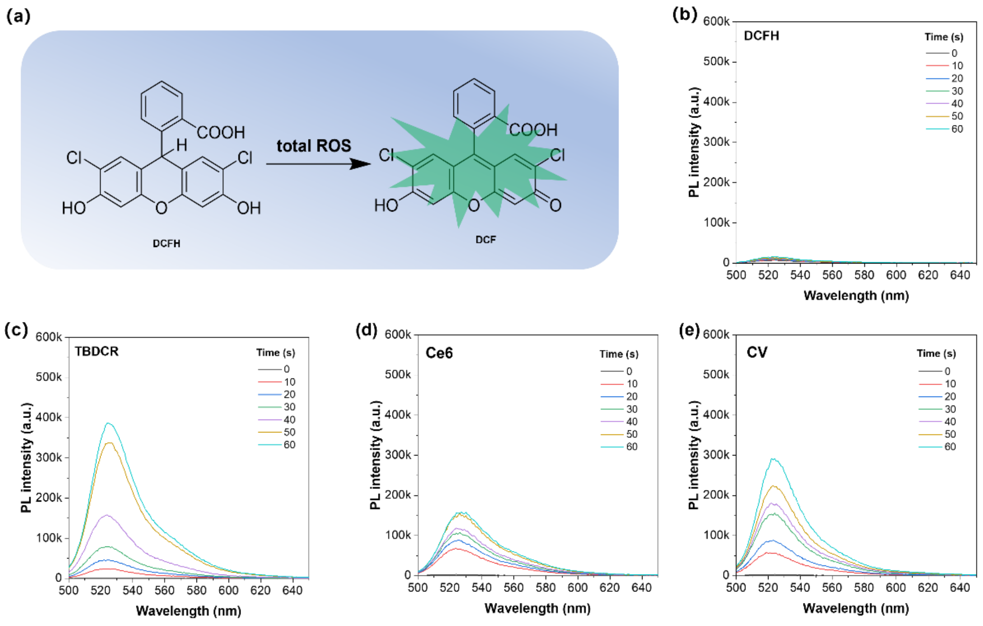

Figure S14. (a) Detection mechanism of DCFH for total ROS. PL spectra of DCFH in the presence of (b) blank, (c) **TBDCR NPs**, (d) Ce6 and (e) CV under the different irradiation time. [PSs] = 10 μM, [DCFH] = 50 μM. White light, 50 mW cm^{-2}.

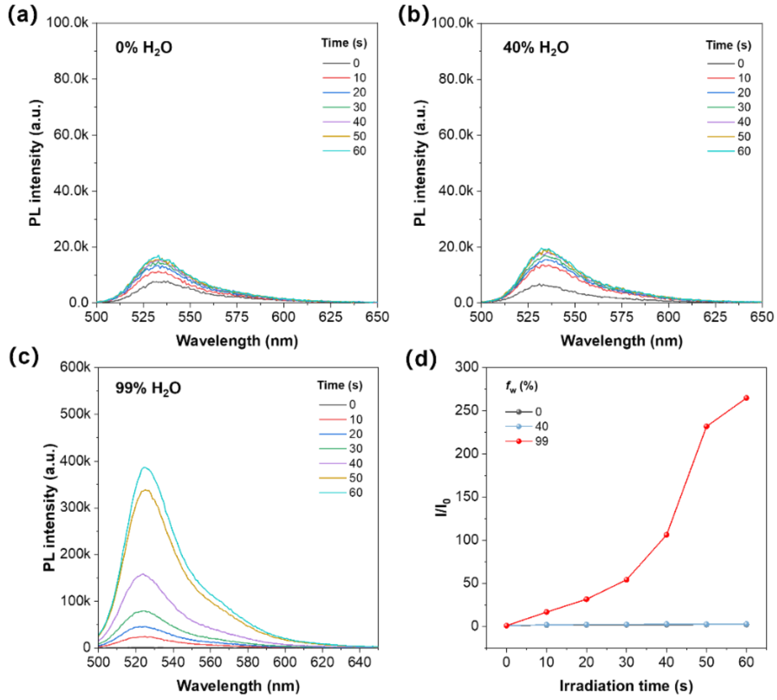

Figure S15. The comparison of ROS production capacity of **TBDCR** (10 μM) at different water fractions of (a) 0%, (b) 40% and (c) 99%, respectively. (d) Access of total ROS generation with the PL enhancement of DCFH (50 μM) for different PSs (10 μM) at different water fraction upon

white light irradiation (50 mW cm^{-2}).

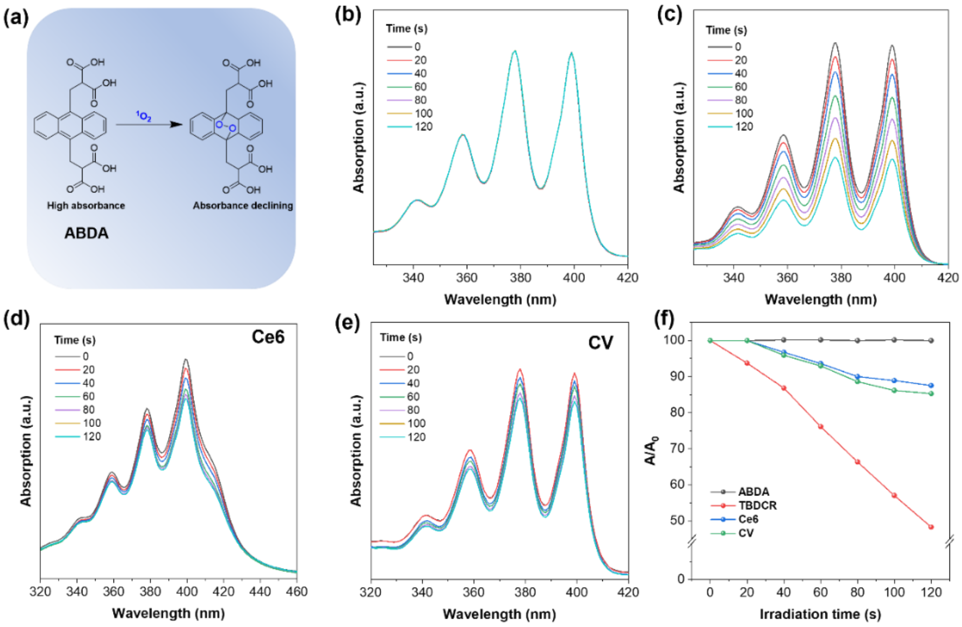

Figure S16. (a) Detection mechanism of ABDA for $^1O_2$. Absorption spectra of ABDA in the presence of (b) blank, (c) **TBDCR NPs**, (d) Ce6 and (e) CV under the different irradiation time. [PSs] = 10 μM, [ABDA] = 50 μM. (f) access of $^1O_2$ generation with the decomposition of ABDA (50 μM) for **TBDCR** (10 μM) under white light irradiation (50 mW cm^{-2}).

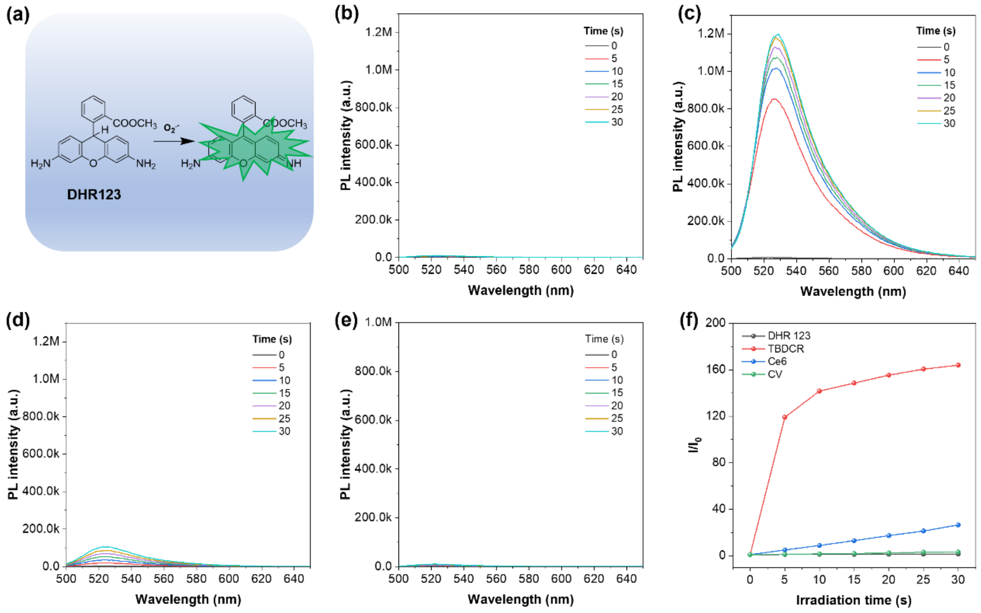

Figure S17. (a) Detection mechanism of DHR 123 for $O_2^{\bullet-}$. PL spectra of HPF in the presence of (b) blank, (c) **TBDCR NPs**, (d) Ce6 and (e) CV under the different irradiation time. [PSs] = 10

μM, [DHR 123] = 20 μM. White light, 50 mW cm^{-2}. (f) access of $O_2^{\bullet -}$ generation with HPF fluorescence enhancement factors.

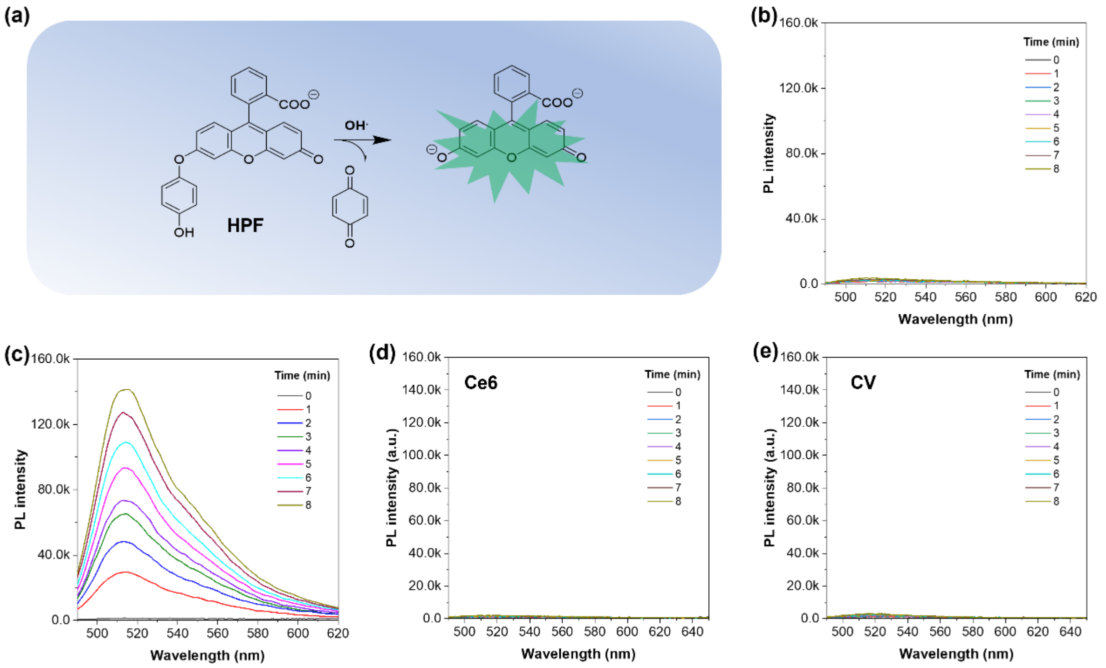

Figure S18. (a) Detection mechanism of HPF for $HO^{\bullet -}$. PL spectra of HPF in the presence of (b) blank, (c) **TBDCR NPs**, (d) Ce6 and (e) CV under the different irradiation time. [PSs] = 10 μM, [HPF] = 50 μM. White light, 50 mW cm^{-2}.

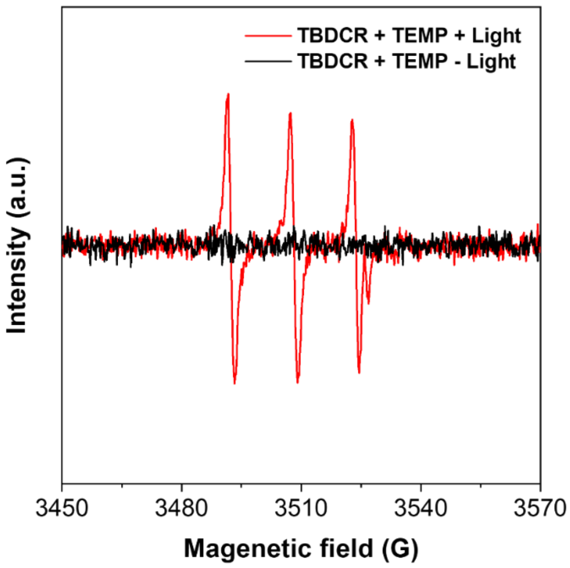

Figure S19. EPR signals of TEMP for free singlet oxygen ($^1O_2$) characterization. [**TBDCR**] = 100 μM, [TEMP] = 50 μM, white light: 100 mW cm^{-2}, irradiation time: 10 min.

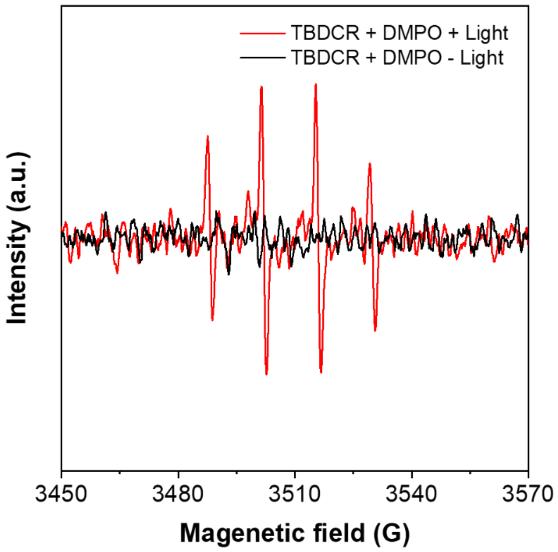

Figure S20. EPR signals of DMPO for free radical ROS ($HO^{\bullet -}$) characterization. [**TBDCR**] = 100 μM, [TEMP] = 50 μM, white light: 100 mW cm^{-2}, irradiation time: 10 min.

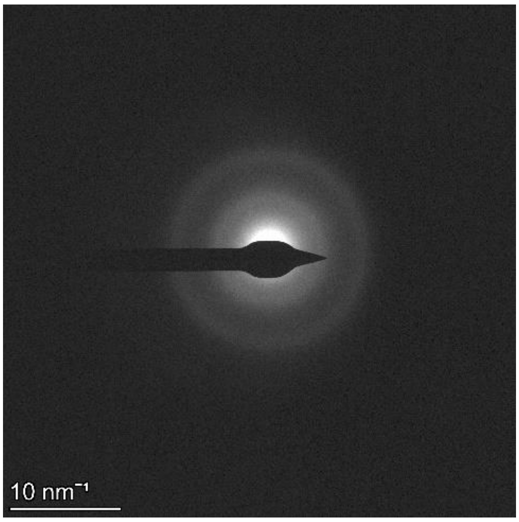

Figure S21. The select area electron diffraction (SAED) result of **TBDCR NPs**.

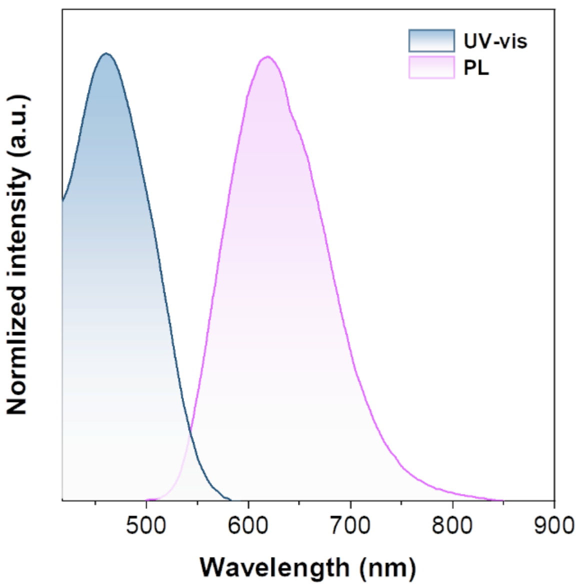

Figure S22. Normalized absorption and PL spectra of **TBDCR NPs** in aqueous solution.

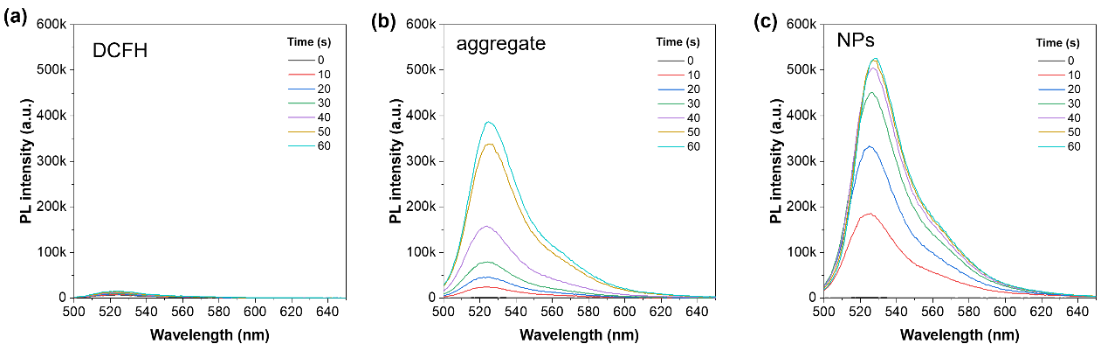

Figure S23. The comparison of ROS generation of (a) black, (b) **TBDCR** aggregate (f_w = 99% in THF/water mixture) and (c) **TBDCR NPs** at the same concentration of **TBDCR** (10 µM).

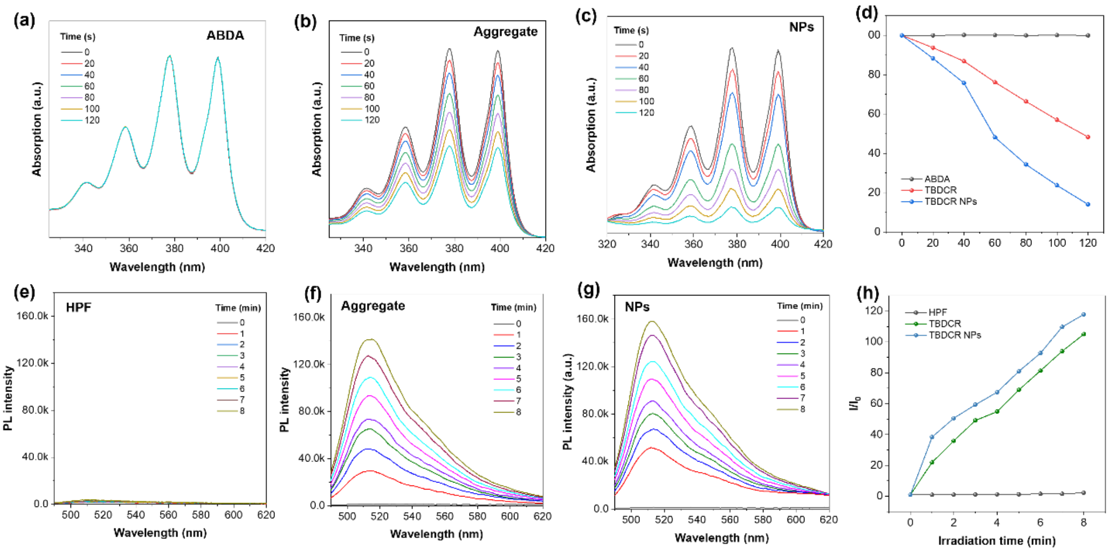

Figure S24. The comparison of $^1O_2$ generation of (a) black, (b) **TBDCR** aggregate (f_w = 99% in THF/water mixture), (c) **TBDCR NPs** at the same concentration of **TBDCR** (10 µM). (d) Access of $^1O_2$ generation with the decomposition of ABDA (50 µM) for **TBDCR** aggregate (f_w =99% in THF/water mixture, 10 µM) and **TBDCR NPs** (10 µM) upon white light irradiation (50 mW cm^{-2}). The comparison of $OH^{\bullet-}$ generation of (e) black, (f) **TBDCR** aggregate (f_w = 99% in THF/water mixture) and (g) **TBDCR NPs** at the same concentration of **TBDCR** (10 µM). (h) Access of $OH^{\bullet-}$ generation with HPF fluorescence enhancement for **TBDCR** aggregate (f_w =99% in THF/water mixture, 10 µM) and **TBDCR NPs** (10 µM) upon white light irradiation (50 mW cm^{-2}).

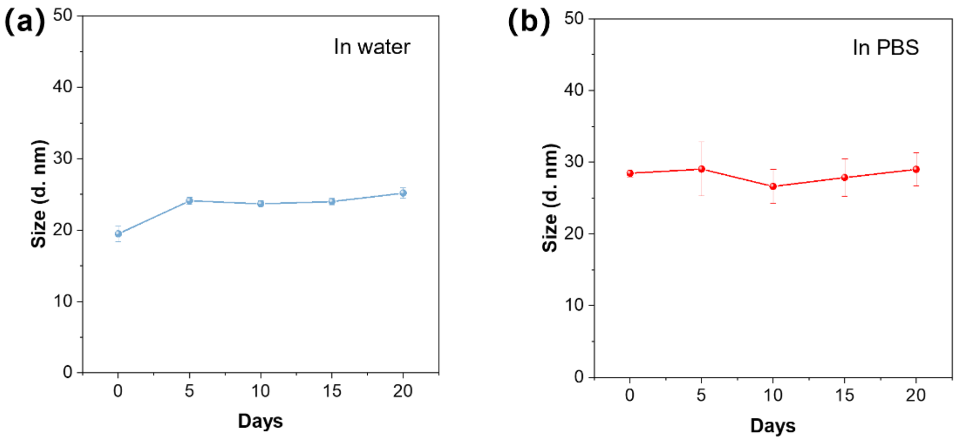

Figure S25. The average hydrodynamic diameter changes of **TBDCR NPs** stored in (a) deionized water and (b) in 1×PBS buffer at 4 □ for 20 days, respectively.

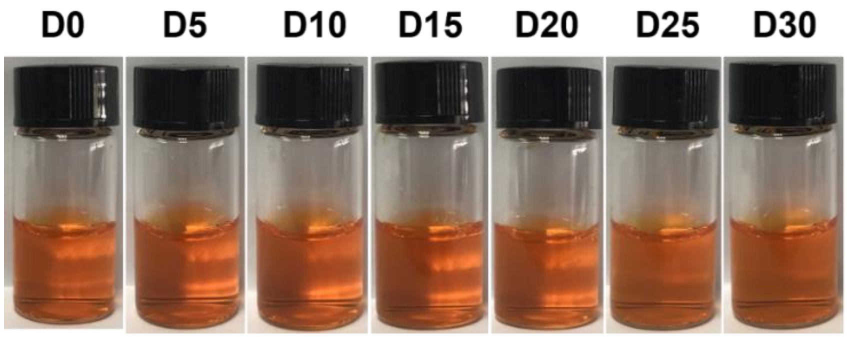

Figure S26. The photographs of the **TBDCR** NPs in deionized water for 30 days storage.

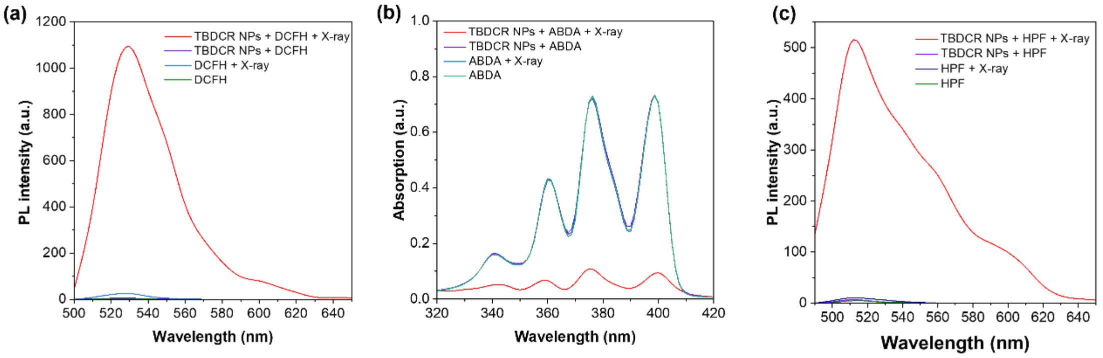

Figure S27. (a) The PL spectra of DCFH in different solutions with or without X-ray irradiation. (b) The absorption spectra of ABDA in different solutions with or without X-ray irradiation. (c) The PL spectra of HPF in different solutions with or without X-ray irradiation. [**TBDCR NPs**] = 40 μg/mL, 4 Gy.

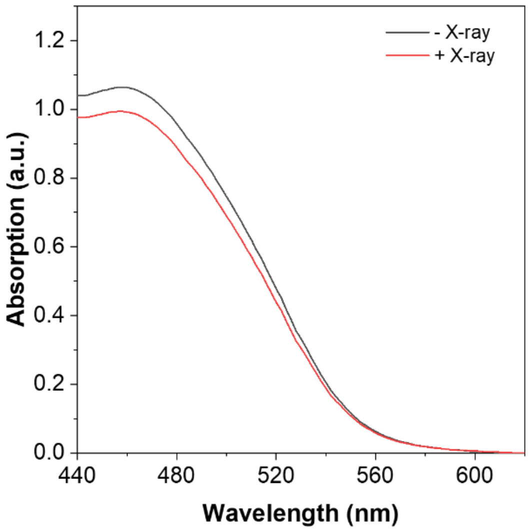

Figure S28. The absorbance spectra of **TBDCR NPs** (40 μg/mL) before and after X-ray irradiation (8 Gy).

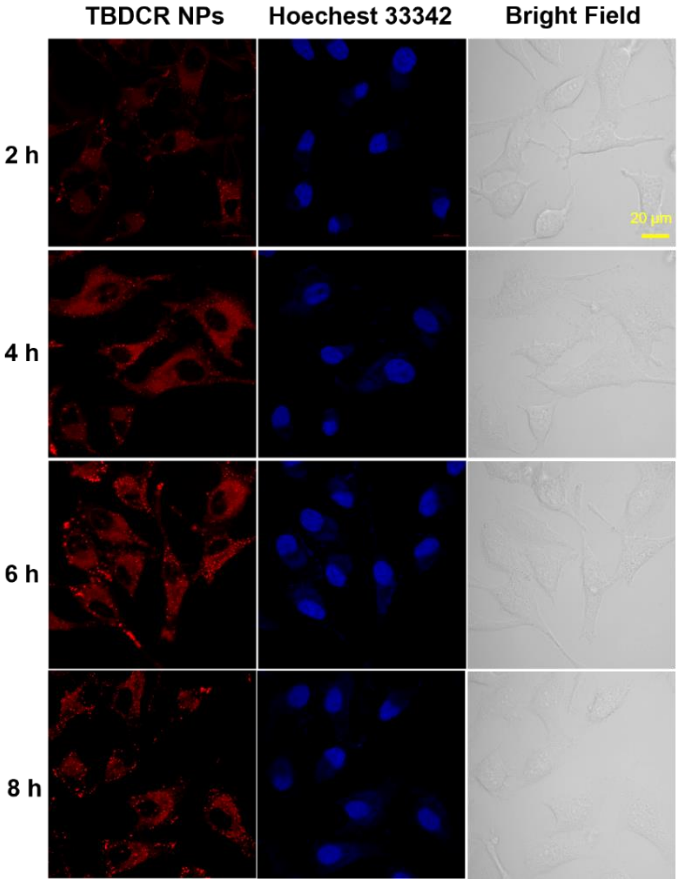

Figure S29. Confocal fluorescence images of cellular uptake of **TBDCR NPs** in Hela cells. **TBDCR NPs**: λ_{ex} = 405 nm, λ_{em} = 550-700 nm; Hoechest 33342: λ_{ex} = 405 nm, λ_{em} = 430-470 nm, scale bars: 20 μm.

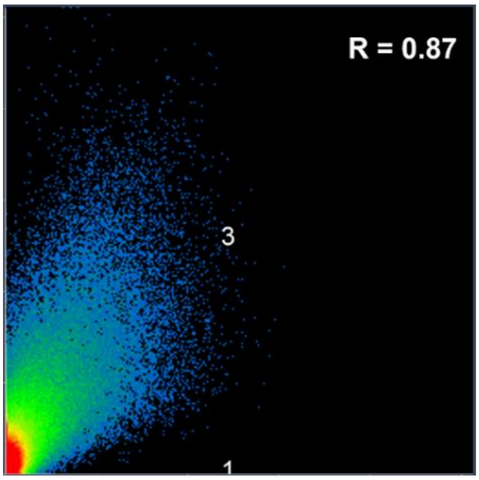

Figure S30. Correlation coefficient of subcellular colocalization images of **TBDCR NPs** and BODIPY 493/503 in Hela cells.

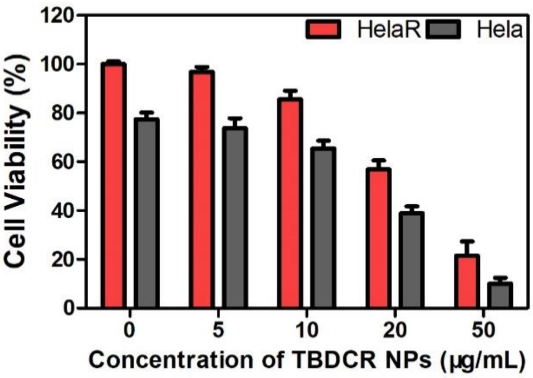

Figure S31. Viabilities of Hela/HelaR cancer cells after treatment with **TBDCR NPs at** varied concentrations and X-ray irradiation doses (8 Gy) under hypoxia (1 % O_2).

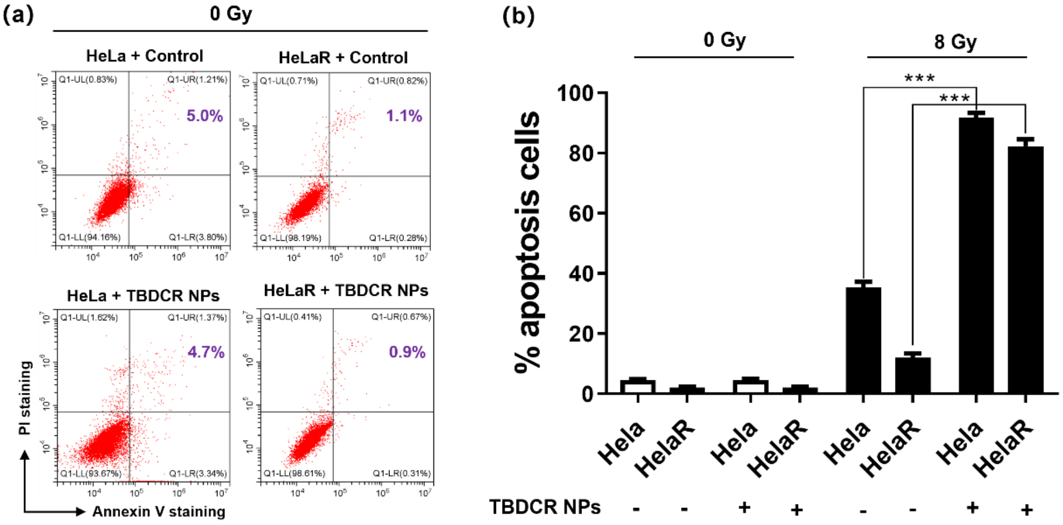

Figure S32. (a) Cytotoxicity of **TBDCR NPs** in an Annexin V apoptosis assay. The Hela/HelaR cells were stained with Annexin V-FITC and propidium iodide. (b) Statistics of cell flowcytometry results under different treatment conditions. Data presented means ± standard deviation (SD). n = 6, ***$p < 0.001$.

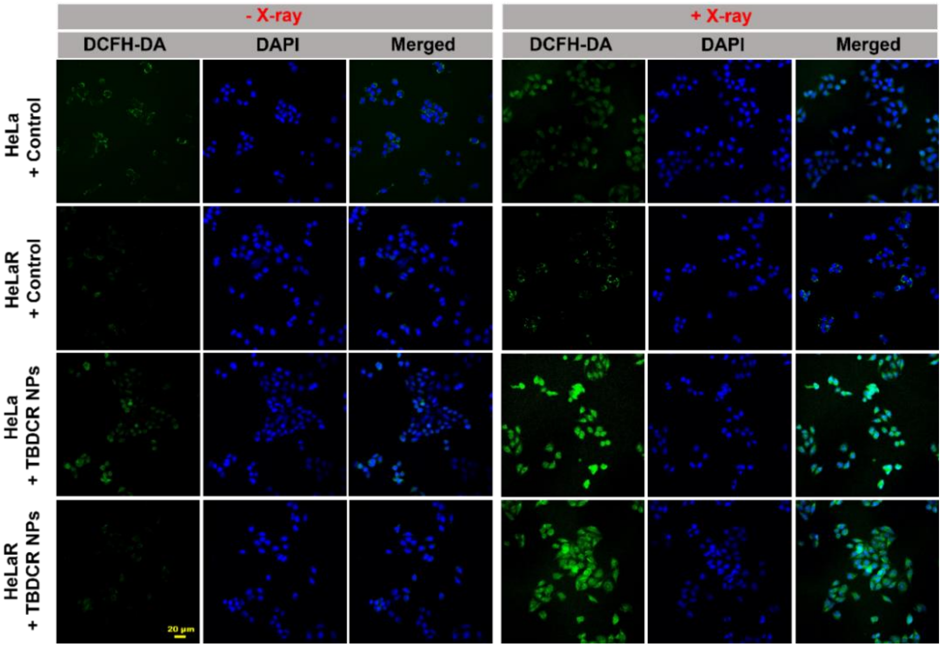

Figure S33. Confocal fluorescence images of DCFH-DA stained Hela or HelaR cells treated with PBS or **TBDCR** NPs and then irradiated with or without X-ray irradiation (8 Gy). Blue: DAPI; Green: DCFH-DA. Scale bar: 20 μm.

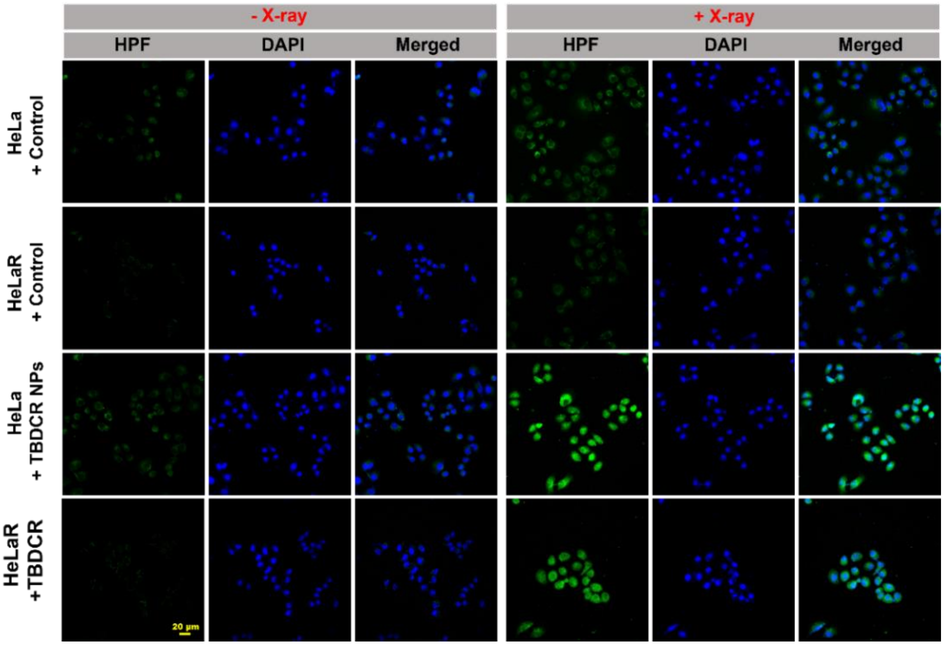

Figure S34. Confocal fluorescence images of HPF stained Hela or HelaR cells treated with PBS, or **TBDCR** NPs and then irradiated with or without X-ray irradiation (8 Gy). Blue: DAPI; Green: DCFH-DA. Scale bar: 20 μm.

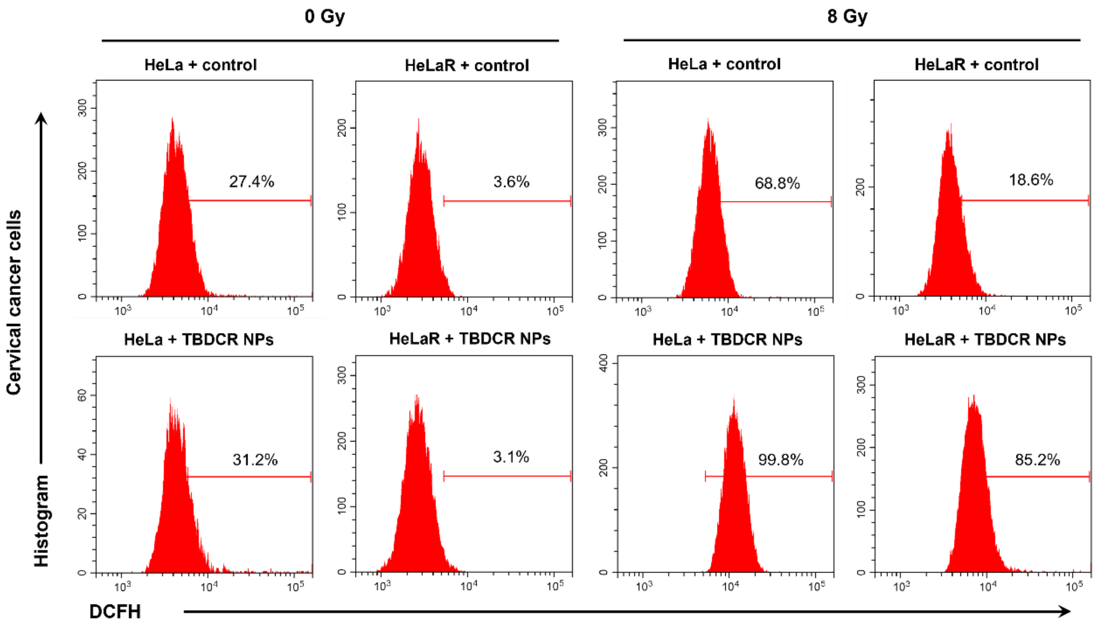

Figure S35. Statistics of cell flow cytometry results of DCFH-DA stained Hela or HelaR cells treated with PBS or **TBDCR** NPs and then irradiated with or without X-ray irradiation (8 Gy).

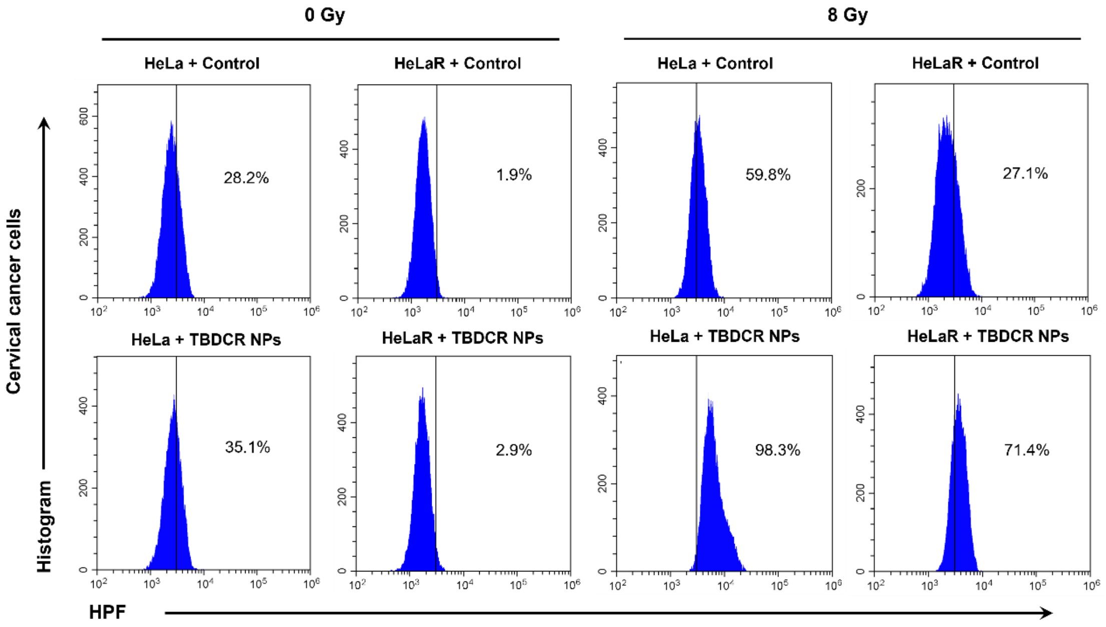

Figure S36. Statistics of cell flow cytometry results of HPF stained Hela or HelaR cells treated with PBS or **TBDCR** NPs and then irradiated with or without X-ray irradiation (8 Gy).

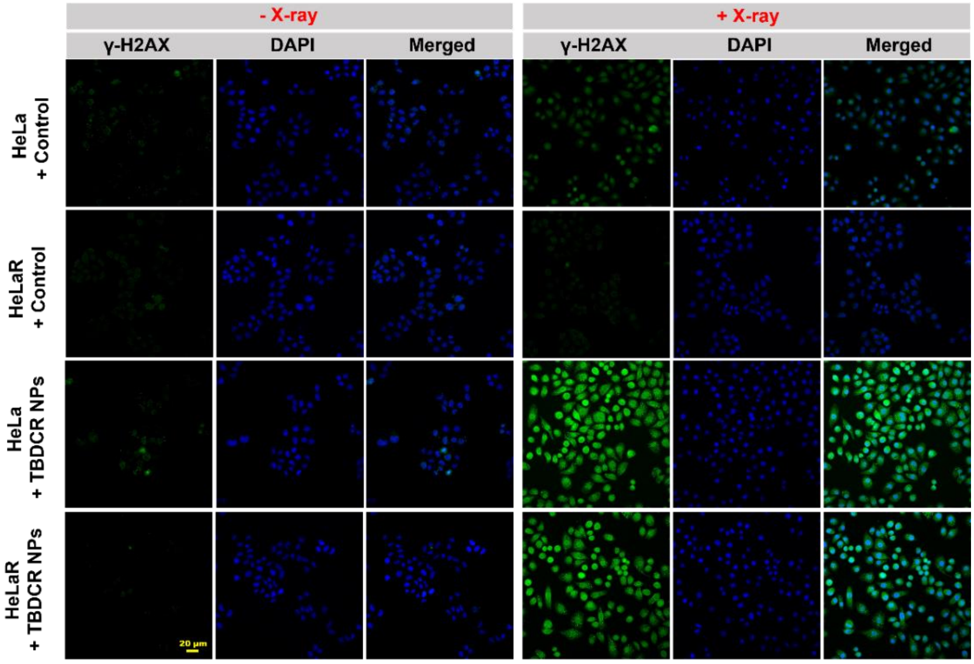

Figure S37. Confocal fluorescence images of γ-H2AX stained Hela or HelaR cells treated with PBS or **TBDCR** NPs and then irradiated with or without X-ray irradiation (8 Gy). Blue: DAPI; Green: γ-H2AX. Scale bar: 20 μm.

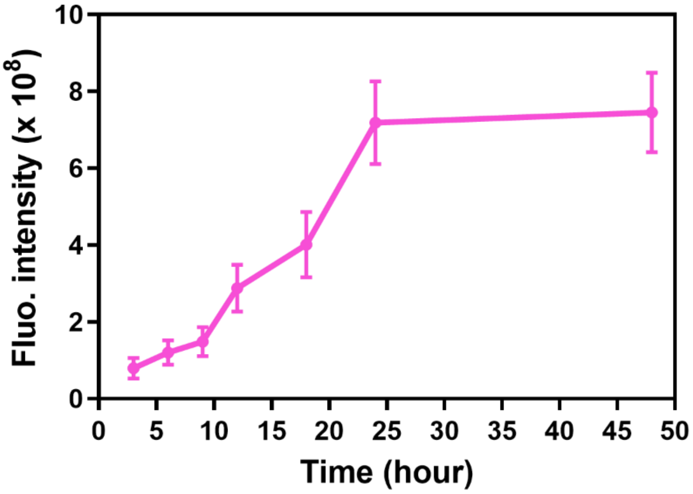

Figure S38. The tumor fluorescence intensity changes at different time points after tail vein injection of **TBDCR NPs**.

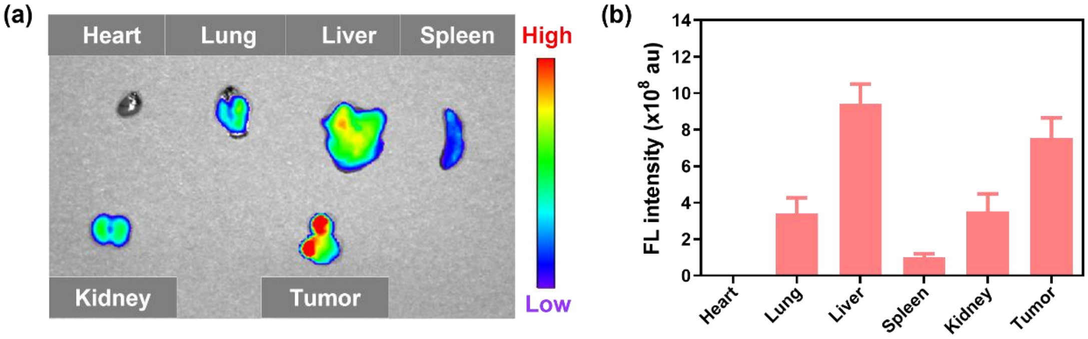

Figure S39. (a) *Ex vivo* fluorescence images of the internal organs of mice sacrificed at 48 h post-injection of **TBDCR NPs**. (b) the corresponding fluorescence intensities of these major organs.

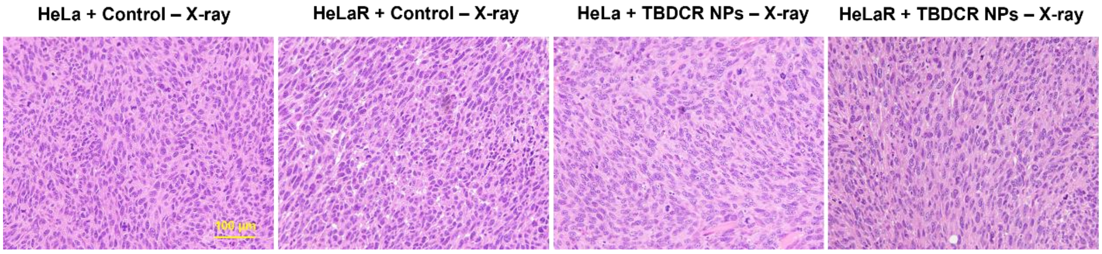

Figure S40. H&E staining of tumor tissues of mice after 42 days of treatment. Scale bar: 100 µm.
